# Corp Regulates P53 in *Drosophila melanogaster* via a Negative Feedback Loop

**DOI:** 10.1371/journal.pgen.1005400

**Published:** 2015-07-31

**Authors:** Riddhita Chakraborty, Ying Li, Lei Zhou, Kent G. Golic

**Affiliations:** 1 Department of Biology, University of Utah, Salt Lake City, Utah, United States of America; 2 Department of Molecular Genetics and Microbiology, College of Medicine, University of Florida, Gainesville, Florida, United States of America; The University of North Carolina at Chapel Hill, UNITED STATES

## Abstract

The tumor suppressor P53 is a critical mediator of the apoptotic response to DNA double-strand breaks through the transcriptional activation of pro-apoptotic genes. This mechanism is evolutionarily conserved from mammals to lower invertebrates, including *Drosophila melanogaster*. P53 also transcriptionally induces its primary negative regulator, Mdm2, which has not been found in *Drosophila*. In this study we identified the *Drosophila* gene *companion of reaper* (*corp*) as a gene whose overexpression promotes survival of cells with DNA damage in the soma but reduces their survival in the germline. These disparate effects are shared by *p53* mutants, suggesting that Corp may be a negative regulator of P53. Confirming this supposition, we found that *corp* negatively regulates P53 protein level. It has been previously shown that P53 transcriptionally activates *corp*; thus, Corp produces a negative feedback loop on P53. We further found that *Drosophila* Corp shares a protein motif with vertebrate Mdm2 in a region that mediates the Mdm2:P53 physical interaction. In Corp, this motif mediates physical interaction with *Drosophila* P53. Our findings implicate Corp as a functional analog of vertebrate Mdm2 in flies.

## Author Summary

Organisms have exquisitely sensitive mechanisms to detect and respond to DNA damage. If DNA damage in a cell can be repaired, then that cell may resume its normal function. In multi-cellular organisms, if a cell cannot repair DNA damage it usually undergoes programmed cell death. This prevents the proliferation of cells with damaged genomes, which might otherwise differentiate incorrectly or proliferate without limit as cancer. In *Drosophila melanogaster* we identified *corp* as a gene that promotes the survival of such cells. Transcription of *corp* is activated by the P53 tumor suppressor protein, known primarily for its induction of cell death in response to DNA damage. Our experiments show that P53 regulates both pro-death and anti-death genes, and that a competition between these opposing factors determines cell fate. We find that *corp* functions by down-regulating P53, establishing a negative feedback loop. In vertebrates an identical mode of regulation is known: P53 up-regulates *Mdm2*, which physically interacts with P53 and is its primary negative regulator. We identified a protein motif on Corp that is shared with Mdm2, and is required for physical interaction between Corp and *Drosophila* P53. These results reinforce and strengthen the similarities of the P53 pathways and their regulation in vertebrates and in *Drosophila*.

## Introduction

When cells encounter damage to their DNA in the form of DSBs, DNA damage response (DDR) pathways are triggered. The ensuing signaling cascades result in cell cycle arrest, induction of DNA repair genes, and in some cases, apoptosis. It is generally thought that, if damage cannot be repaired, cells will undergo apoptosis rather than continue to divide and propagate a damaged genome. If cells with irreparable damage do survive and proliferate, it can result in widespread genomic instability, creating an early state in the progress towards cancer [1–3]. In *Drosophila melanogaster*, most cells undergo apoptosis in response to irreparable DNA damage, but a few cells escape, continue to divide and exhibit genomic instability [4–6]. In our current study, we aimed to investigate the genetic mechanisms that allow cells to survive in the presence of irreparably damaged DNA.

One of the key players that controls the fate of a cell following DNA damage is the tumor-suppressor encoded by the *p53* gene, which is found to be mutated in most human cancers [7]. In response to DNA damage, the ATM kinase (encoded by *tefu* in *Drosophila*) phosphorylates Chk2 (encoded by *lok*), which in turn phosphorylates and activates P53 [8–10]. Activated P53 is primarily a transcriptional regulator that promotes or inhibits the expression of a large number of target genes that encode a variety of cellular functions such as DNA repair, cell cycle arrest and apoptosis [11–17]. A cell that detects damage and engages the P53 damage response pathway may either repair the damage or experience senescence or death [1–3,18]. Humans that lack one copy of *p53* are prone to develop cancers, and *p53* knockout mice develop cancers at an increased rate [1–6,19,20]. Similarly, *p53*-null *Drosophila* fail to eliminate cells with a DSB in their genome [5,7,20–23].

The *Mdm2* gene is a prominent target of P53 in mammals. It encodes a ubiquitin ligase that negatively regulates P53 and promotes its degradation, constituting a negative feedback loop [8–10,24,25]. Mediation of apoptosis by P53 is highly conserved throughout metazoa, including *Drosophila melanogaster* [11–18,21–23,26]. However, apart from DNA repair genes, targets of p53 that antagonize apoptosis have yet to be reported in flies and no homolog of *Mdm2* has been identified.

Here, we report the identification of a gene, *companion of reaper* (*corp*), whose overexpression mimics the effects of *p53* mutants in the soma and the germline. Our experiments indicate that Corp negatively regulates P53 protein levels. The *corp* gene has been previously identified as a transcriptional target of P53 [14,15,27], thus Corp acts on P53 in a negative feedback loop. Furthermore, Corp exhibits similarity to Mdm2 in a region essential for the Mdm2-P53 interaction, and we find that this region of Corp mediates a physical interaction with *Drosophila* P53. These similarities lead us to conclude that *corp* encodes a functional analog of vertebrate Mdm2 in flies and strengthens the similarities between the regulation and the functions of P53 in *Drosophila* and mammals.

## Results

### Corp suppresses tissue ablation resulting from DNA damage

**Fig 1:**
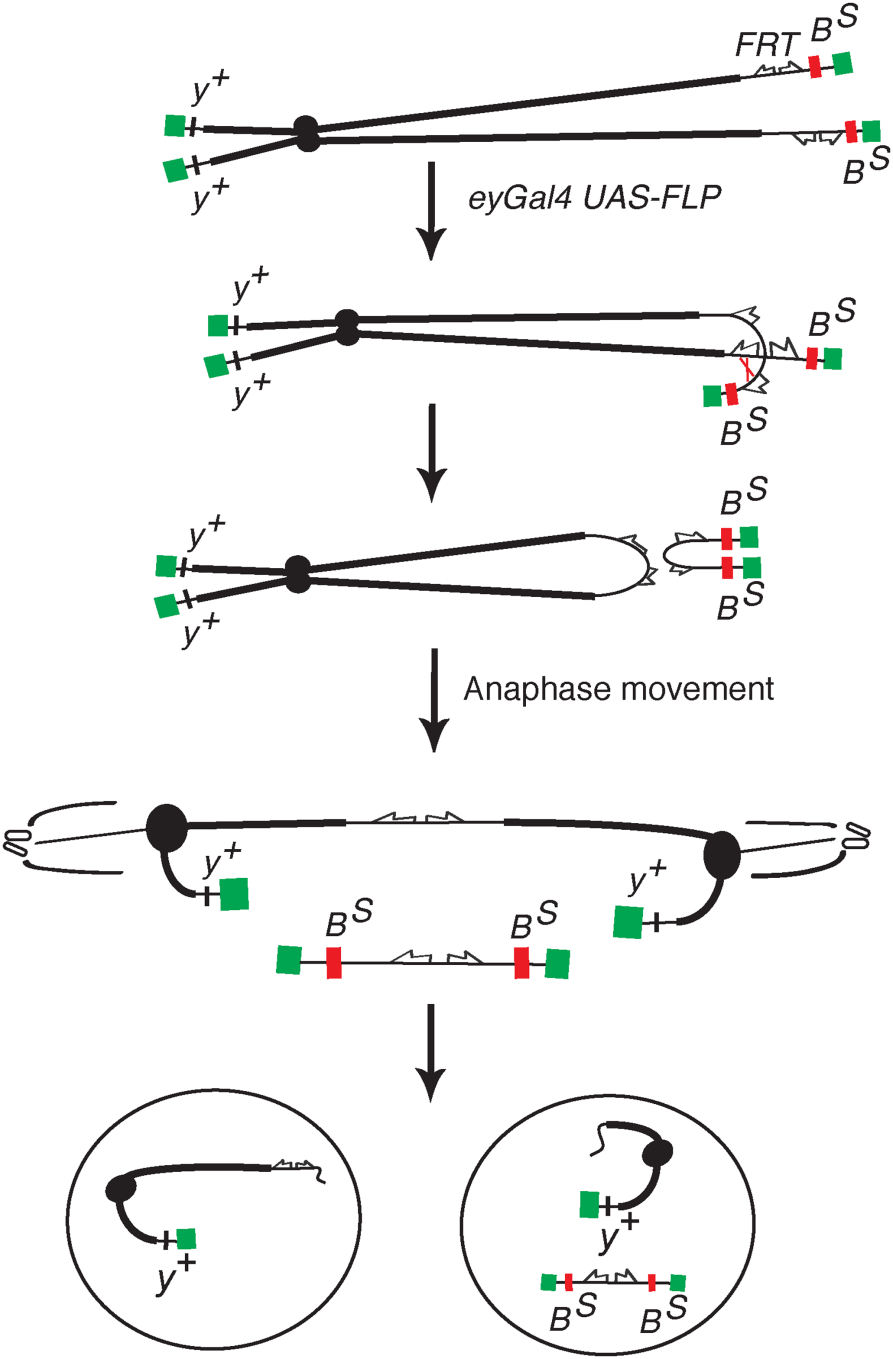
The *DcY(H1)* chromosome and BARTL assay. Chromosome breakage and telomere loss in the BARTL assay. The *DcY(H1)* chromosome (drawn as sister chromatids in G2) is a *Y* chromosome that carries *y* ^*+*^ on the short arm and dominant *B* ^*S*^ on the long arm. Inverted *FRTs* are inserted proximal to *B* ^*S*^. The centromere is represented as a solid black circle, the telomeres as green rectangles, *B* ^*S*^ gene as a red rectangle and inverted *FRTs* as half-arrows. When FLP mediates unequal sister chromatid exchange between inverted *FRT*s a dicentric and an acentric chromosome are produced. In the subsequent mitotic anaphase the dicentric chromosome is pulled to opposite poles and usually breaks. Each daughter cell receives a chromosome with a single broken end and one or both daughter cells lose the *B* ^*S*^-containing acentric fragment.

**Fig 2:**
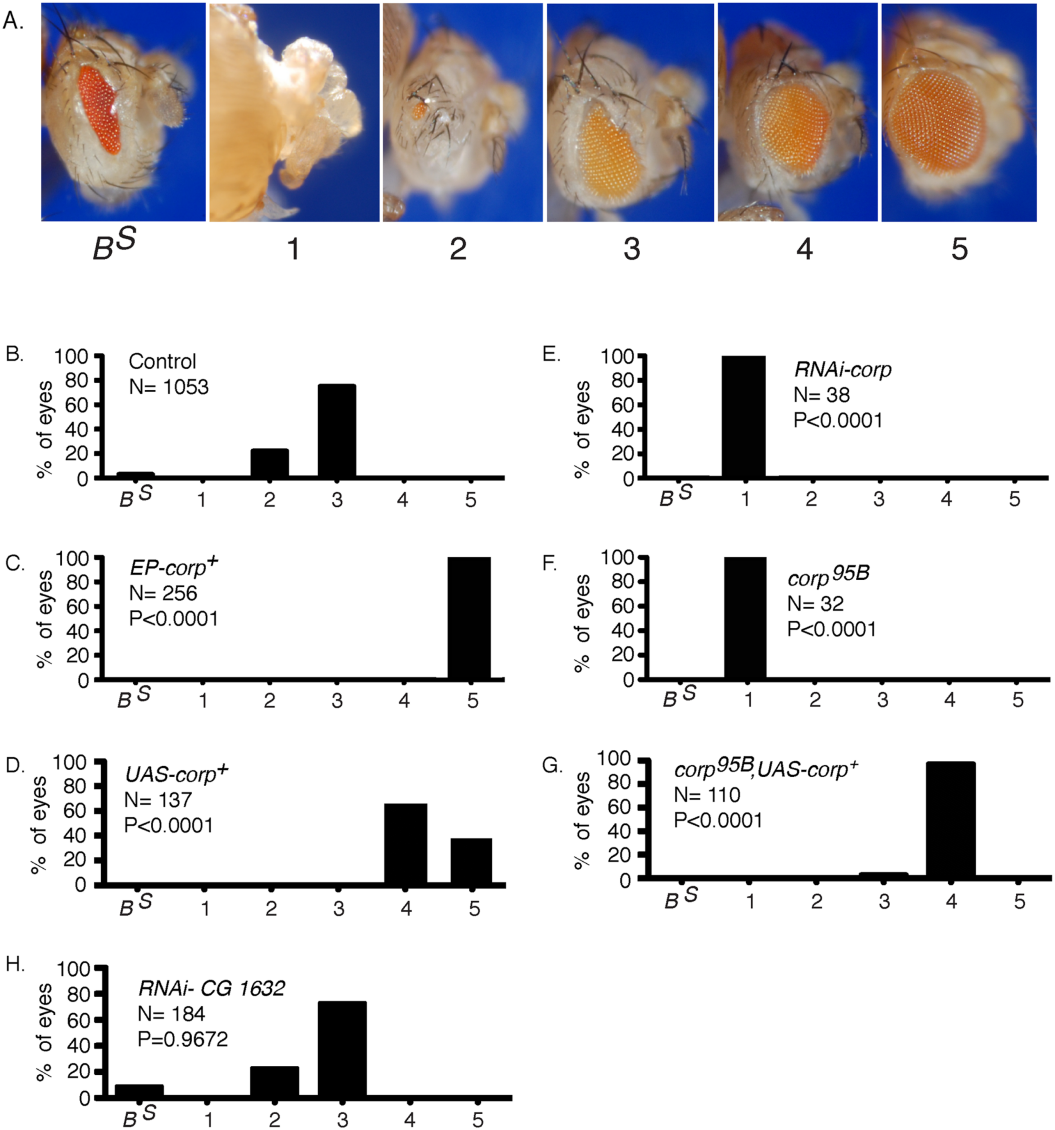
Overexpression of *corp* ^*+*^ suppresses the BARTL phenotype. (A) The range of eye phenotypes observed in the assay. The *B* ^*S*^ phenotype of *H1* control males is shown at the left. When FLP is expressed (*ey>FLP*), the phenotypes can range from headless pharates (category 1) to adults with a fully developed wildtype eye (category 5). The distribution produced (B) in control males; (C) in males carrying *P{EPgy2}EY03495*, referred to as *EP-corp* ^*+*^; (D) by inclusion of a *UAS-corp* ^*+*^ transgene; (E) with RNAi-mediated knockdown of *corp*; (F) in *corp* ^*95B*^ mutants; (G) with rescue of the *corp* ^*95B*^ phenotype by expression from the *UAS-corp* ^*+*^ transgene; and, (H) with RNAi-mediated knockdown of *CG1632*. N represents number of eyes scored for each genotype. Each *P* value represents comparison with the wildtype control, shown in B. Genotypes were: (B) *y w/H1; eyGal4 UAS-FLP/+*; (C) *y w EP-corp* ^*+*^ */H1; eyGal4 UAS-FLP/+*; (D) *y w/H1; eyGal4 UAS-FLP/+; UAS-corp* ^*+*^ */+*; (E) *y w/H1; eyGal4 UAS-FLP/RNAi-corp*; (F) *y w corp* ^*95B*^ */H1; eyGal4 UAS-FLP/+*; (G) *y w corp* ^*95B*^ */H1; eyGal4 UAS-FLP/+; UAS-corp* ^*+*^ */+*; (H) *y w/H1; eyGal4 UAS-FLP/RNAi-CG1632*.

The previously described BARTL (*Bar* and Telomere Loss) assay [20] was used to screen for insertions of a *P*-element mis-expression element [28] that modify the eye phenotype resulting from the production of an irreparable DNA DSB. In brief, a combination of *eyeless-Gal4* (*eyGal4*) and *UAS*-*FLP* is used to drive FLP recombinase expression in proliferating cells of the eye throughout development [29–31]. These flies also carry a *Y* chromosome with inverted *FRT* repeats (*DcY(H1)*, or simply *H1*). Recombination between *FRT*s in inverted orientation on sister chromatids produces dicentric chromosomes which break in the subsequent mitotic division, delivering a chromosome with a single broken end to each of the two daughter cells (Fig 1). This results in substantial P53-mediated apoptosis and produces flies with characteristic small and rough eyes (Fig 2B). By introducing an *EP* transposon insertion, which carries *UAS* elements that can drive expression of a neighboring gene, the flies’ eyes may become larger or smaller, indicating that the *EP* element in question modifies the fate of cells in these eyes. We identified one such *EP* insertion (*P{EPgy2}CG1632* ^*EY03495*^) that produced nearly wildtype eyes in the BARTL assay (Fig 2C). This insertion was ideally placed to drive expression of the *corp* ^*+*^ gene (*CG10965*). By qRT-PCR we confirmed that when Gal4 induces the *P{EPgy2}CG1632* ^*EY03495*^ element (hereafter referred to as *EP-corp* ^*+*^) it drives overexpression of *corp* ^*+*^ (S1 Fig). We also constructed a *UAS-corp* ^*+*^ transgene, and found that its effect was nearly identical to that produced by *EP-corp* ^*+*^ (Fig 2D).

When we tested RNAi-mediated knockdown of *corp* in the BARTL assay the opposite result was obtained: the eye was completely ablated (Fig 2E; *n*. *b*., *ey* expression extends beyond the eye proper, accounting for, in some cases, nearly complete ablation of the head). A *corp* mutant was also generated by imprecise excision of a *P* element located in the 5’ region of the gene. This mutant, *corp* ^*95B*^ (S2 Fig), is viable in homozygous condition and without obvious phenotype on its own. However, like RNAi-mediated *corp* knockdown, *corp* ^*95B*^ completely ablates the eye in the BARTL assay (Fig 2F). This effect can be rescued by the *UAS-corp* ^*+*^ transgene (Fig 2G).

To verify that the gene *CG1632*, in whose intron *corp* is located and which is transcribed opposite to *corp* (S2 Fig), is not responsible for these phenotypes we tested RNAi-mediated knockdown of *CG1632* in the BARTL assay and found no significant change (Fig 2H). Combined with the observations that a *UAS-corp* ^*+*^ construct produces the same phenotype as *EP-corp* ^*+*^ and that RNAi against *corp* has the opposite effect, it is clear that the effects we observe owe to *corp* and not *CG1632*.

If *EP-corp* ^*+*^ was not induced by Gal4, and *eyFLP* was instead used to produce dicentric chromosomes in the eye, we found that the *EP-corp* ^*+*^ insertion produced slightly larger eyes than the wildtype control, suggesting that the *EPgy2* insertion by itself may have slightly elevated *corp* expression (S3B Fig). This effect, though statistically significant, is small and only *eyGal4*-mediated *corp* ^*+*^ overexpression can generate the wildtype-like eye phenotype in the BARTL assay (Fig 2C and 2D).

To determine whether *corp* has any influence in the absence of DNA damage we examined wild type or *B* ^*S*^ flies carrying *EP-corp* ^*+*^, induced or uninduced, and flies carrying the *corp* ^*95B*^ mutant, but without the induction of dicentric chromosomes. There was no change in eye phenotype in any of these cases, indicating that the effects of altered *corp* ^*+*^ expression are seen only after DNA damage (S3C–S3G Fig).

*EP-corp* ^*+*^-mediated rescue of the eye is not confined to males, or to effects produced by the *Y* chromosome. We generated *XXY* females carrying *eyGal4*, *UAS-FLP* and the *DcY(H1)* chromosome and found that *EP-corp* ^*+*^ produced almost wildtype eyes, similar to its effect in males (S3H and S3I Fig). Additionally, we found that *corp* ^*+*^ overexpression ameliorated the reduction in eye size produced by dicentric induction on chromosome *3* (S3J and S3K Fig). Therefore, the effect of *corp* ^*+*^ overexpression is independent of the sex of the fly or the particular chromosome experiencing damage.

### Extant *corp* polymorphisms are functionally indistinguishable

We sequenced the *corp* genomic regions from five laboratory strains and found two allelic variants of *corp* that differed by 7 nucleotide changes. Four nucleotide changes were found in the second exon: two are silent mutations (C840T and C891T) and two (T725A and T873G) encode different amino acids (L96H and L145M). These alternate amino acids are also present as polymorphisms in wildtype isolates of *D*. *melanogaster* and other *Drosophila* species (http://www.dpgp.org). There were also three single nucleotide differences (C277T, A398C and A407T) in introns.

The *UAS-corp* ^*+*^ transgene that we constructed and tested (as mentioned previously) carries the canonical version, as found in *Canton* ^*S*^. The *EP-corp* ^*+*^, *y w and w* ^*1118*^ strains all carry the variant allele that differs from the reference *Canton* ^*S*^ strain. Because the overexpression of either allele produces a similar large eye phenotype in the BARTL assay (Fig 2C and 2D), we conclude that both alleles function similarly, and that the two amino acid differences have, at most, minor effects.

### *corp* ^*+*^ suppresses DNA damage-induced apoptosis in the soma

**Fig 3:**
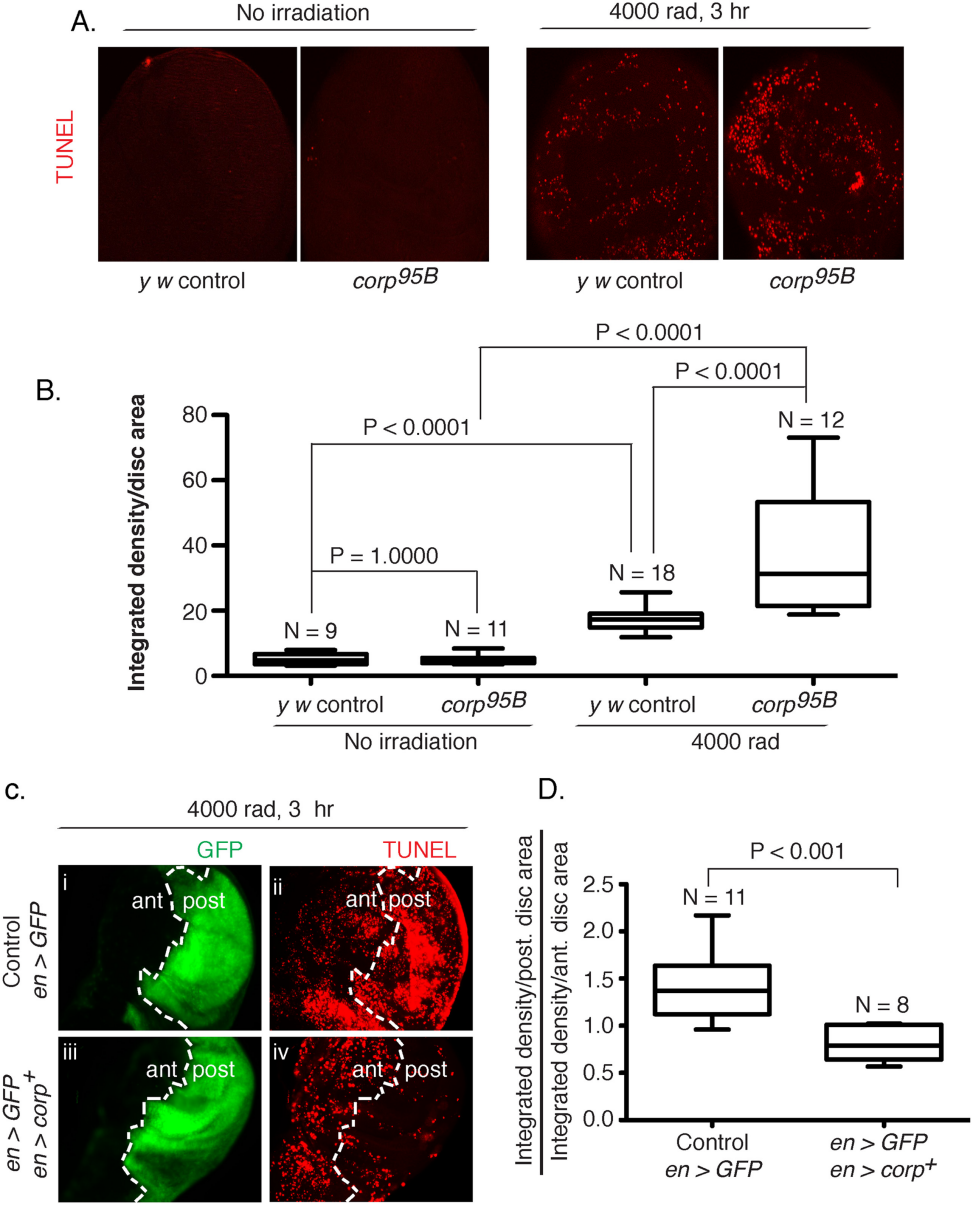
Corp inhibits DNA damage-induced apoptosis in somatic tissue. (A-B) The effect of the *corp* ^*95B*^ mutant assayed by TUNEL staining of wing discs from third instar larvae with or without exposure to irradiation (IR). Very little apoptosis was detected in control or mutant genotypes without irradiation, but staining is greatly increased 3 hours after 4000 rads of irradiation, and *corp* ^*95B*^ mutants exhibit significantly more staining than the *y w* control. Images were captured with a 20X objective at 800 ms shutter speed. (B) Quantitation. Intensity of TUNEL staining is measured as Integrated density (InD = total pixel intensity in the selected region) per unit area of wing disc for *y w* control and *corp* ^*95B*^ mutant, with and without irradiation. N represents total number of discs used for quantification. (C-D) The effect of *corp* ^*+*^ overexpression on cell death is assayed by TUNEL staining of third instar wing discs 3 hours after exposure to 4000 rads of IR. The white broken line marks the boundary between the anterior (ant) and posterior (post) compartments of the disc, based on *engrailed*-driven GFP fluorescence that marks the posterior compartment (i and iii). (i, ii) The staining pattern in wildtype control. (iii, iv) Staining in discs where *engrailed* is driving *EP-corp* ^+^ overexpression in posterior compartment. TUNEL staining is dense in wildtype larvae in the posterior segment (ii), but is greatly reduced with *corp* ^*+*^ overexpression (iv). All images were taken with a 20X objective at 500 ms shutter speed. (D) Quantitation. InD per unit area was measured individually for the anterior and posterior compartment of each disc and the Y-axis is denoted as a ratio of InD per unit area of the posterior compartment to that of the anterior compartment. Genotypes tested are indicated on the X-axis. N represents total number of discs used for quantification.

One mechanism by which Corp could affect eye size in the BARTL assay is through the suppression of apoptosis, thereby allowing survival and proliferation of cells that would otherwise die. To test this we stained wing imaginal discs for apoptotic cells after treatment with ionizing radiation (IR). Untreated discs show similar low rates of apoptosis in both the *y w* controls and the *corp* ^*95B*^ mutants, but IR-induced apoptosis was significantly enhanced in *corp* ^*95B*^ mutants (Fig 3A and 3B). We then examined the effect of *corp* ^*+*^ overexpression. An *engrailed-Gal4* element [32] was used in combination with *EP-corp* ^*+*^ to drive expression in the posterior compartment of wing discs, which was also marked by co-induction of *UAS-GFP*. Apoptosis was significantly reduced by *corp* ^*+*^ overexpression (Fig 3C and 3D). These results show that Corp is a potent negative regulator of apoptosis following DNA damage in the soma.

### Overexpression of *corp* ^*+*^ restricts the transmission of healed chromosomes through the germline

In the male germline, broken dicentric chromosomes may be healed by *de novo* telomere addition [33,34]. With *DcY(H1)* these healed chromosomes (denoted *FrY*) may be detected in testcrosses to *y w* females by the loss of the dominant *B* ^*S*^ marker that lies distal to the inverted *FRT*s (*i*.*e*., by the generation of *Bar* ^*+*^ sons). To assess the effect of *corp* on the transmission of broken-and-healed chromosomes we induced expression of FLP by heat shock (*70FLP10*) during the first 24 hours of development and used *nanosGal4* to drive germ cell-specific overexpression of *corp* ^*+*^. Overexpression of *corp* ^*+*^ blocked transmission of *FrY* chromosomes (Table 1).

**Table 1:** Effect of *corp* on transmission of broken-and-healed chromosomes through the male germline.

Genotype	N^a^	Sterility (%)^b^	*FrY* sons	*Y* sons	Fragment Ratio^c^	Daughters
*y w/H1; 70FLP10/+*	317	55	6401	14411	0.31	25011
*y w EP-corp* ^*+*^ */H1*; *70FLP10/+; nanosGal4/+*	247	51	7	9801	0.001 *P*<0.0001	11611
*y w/H1; nanosGal4 UAS-FLP/+*	247	14	11998	2425	0.83	16675
*y w EP-corp* ^*+*^ */H1; nanosGal4 UAS-FLP/+*	174	30	2180	7573	0.22 *P*<0.0001	11386

^*a*^ N, number of males testcrossed.

^*b*^ Percentage of tested males that were sterile.

^*c*^ Fragment ratio is calculated as *FrY* sons/total sons. *P* values were determined with the Mann-Whitney test using Fragment Ratios of individual males. Each *P* value represents comparison with the row immediately above in this table.

We also drove *corp* ^*+*^ and *FLP* expression specifically in the germline using *nanosGal4 (EP-corp* ^*+*^ *; UAS-FLP nanosGal4)* and again observed a large decrease in *FrY* transmission relative to males with unaltered *corp* expression (Table 1), confirming that *corp* ^*+*^ inhibits transmission of broken-and-healed chromosomes through the male germline.

### The relationship between *corp* and *p53*

Though it seems surprising that *corp* ^*+*^ overexpression produces dissimilar phenotypes in the soma (survival and proliferation of cells with broken chromosomes) vs. the germline (elimination of cells with broken chromosomes), there is precedent: the *p53* ^*5A-1-4*^ loss of function mutation acts similarly. Homozygous *p53* ^*5A-1-4*^ flies have almost wildtype eyes in the BARTL assay [20], but strongly reduced transmission of broken-and-healed chromosomes through the male germline [35]. This similarity suggests a functional relationship between *corp* and *p53*.

**Fig 4:**
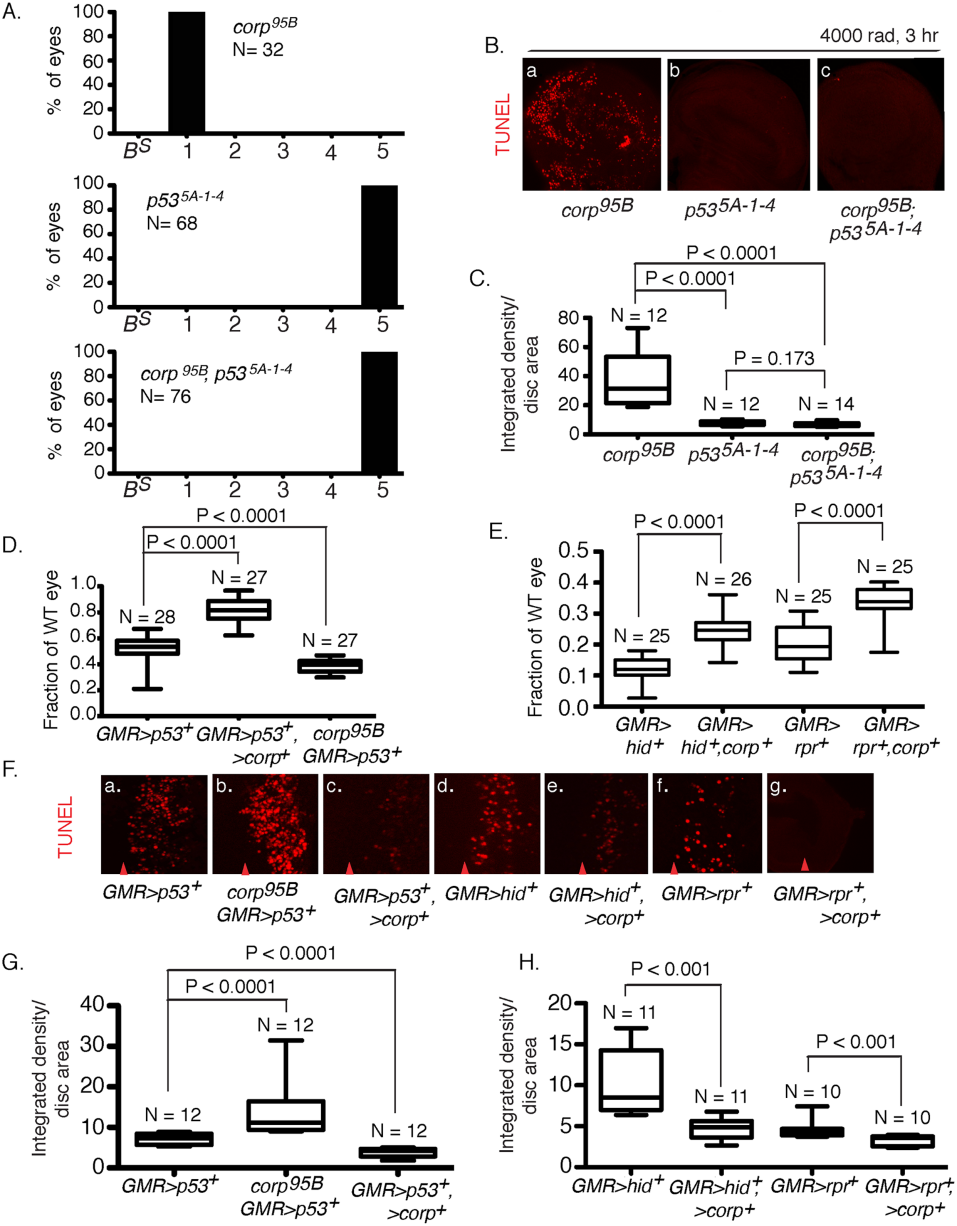
Interaction of *corp* and *p53*. (A) The *p53* ^*5A-1-4*^ mutation is epistatic to *corp* in the BARTL assay. The *corp* ^*95B*^ mutation produces headless flies in the BARTL assay (reproduced from Fig 2), while *p53* ^*5A-1-4*^ has the opposite effect, producing flies with wildtype eyes. The double mutant is indistinguishable from the *p53* ^*5A-1-4*^ single mutant. N represents the number of eyes or headless pharates scored. Genotypes used were *y w corp* ^*95B*^ */H1; eyGal4 UAS-FLP*, *or y w/H1; eyGal4 UAS-FLP/+; p53* ^*5A-1-4*^, or *y w corp* ^*95B*^ */H1; eyGal4 UAS-FLP/+; p53* ^*5A-1-4*^. (B-C) The *p53* ^*5A-1-4*^ mutation suppresses elevated apoptosis observed in *corp* ^*95B*^ mutants. TUNEL staining of wing discs following irradiation shows that apoptosis is highly reduced in *p53* ^5A-1-4^ mutants, compared to intense cell death in *corp* ^*95B*^ mutants (reproduced from Fig 3A). *corp* ^*95B*^ *; p53* ^5A-1-4^ double mutants also show reduced cell death, similar to *p53* ^5A-1-4^ single mutants. Wing discs were TUNEL stained 3 hours after 4000 rads of irradiation. (C) Quantitation. Intensity of TUNEL staining is expressed as integrated density per unit area of the wing disc. (D) Overexpression of *corp* ^*+*^ suppresses the apoptotic phenotype caused by overexpression of *p53* ^*+*^. Eye sizes were measured from flies that overexpressed *p53* ^*+*^, and that also overexpressed *corp* ^*+*^ or were *corp* ^*95B*^ mutants. The Y-axis indicates eye size normalized to wildtype eyes. (F;a-c, G) Overexpression of *corp* ^*+*^ inhibits cell death caused by overexpression of *p53* ^*+*^. *GMR* drives *p53* ^*+*^ expression to produce cell death posterior to the morphogenetic furrow, assayed here by TUNEL staining, The *corp* mutant greatly enhances TUNEL staining while *corp* ^*+*^ overexpression reduces TUNEL staining. Genotypes are as indicated. All images were taken with a 40X objective. (G) TUNEL staining is quantified as InD/area of eye disc posterior to morphogenetic furrow. (E) *corp* ^*+*^ overexpression suppresses the cell-death phenotypes mediated by *hid* ^*+*^ or *reaper* ^*+*^ overexpression. *GMR-Gal4* was used to drive *corp* ^*+*^, *hid* ^*+*^ and *reaper* ^*+*^ overexpression. The Y-axis represents eye size as in D. (F;d-g, H) *corp* ^*+*^ overexpression inhibits apoptosis caused by *hid* ^*+*^ or *reaper* ^*+*^ overexpression. *GMR-Gal4* was used to drive *corp* ^*+*^, *hid* ^*+*^ and *reaper* ^*+*^ overexpression, to produce cell death posterior to the morphogenetic furrow. All images were taken with a 40X objective. (H) Staining is quantified as InD/area, as described above. Red arrowheads in part F indicate approximate position of the morphogenetic furrow in each image. Posterior is to the right. N, the number of eyes or imaginal discs measured for quantification.

To explore this relationship we generated *corp* ^*95B*^ *; p53* ^*5A-1-4*^ double mutants and examined them using the BARTL assay. We found that *p53* is epistatic to *corp*, with the double mutant producing almost wildtype eyes (Fig 4A). In a direct measurement of apoptosis in wing discs following treatment with ionizing radiation (IR) we observed the same epistatic relationship: *p53* ^*5A-1-4*^ suppressed the elevated apoptosis produced by *corp* ^*95B*^, and the double mutant was indistinguishable from the *p53* single mutant (Fig 4B and 4C).

In a complementary experiment, we tested the effect of simultaneously overexpressing *corp* ^*+*^ and *p53* ^*+*^. When *GMR-Gal4* drives *p53* ^*+*^ overexpression in the developing eye, the adults that eclose have very small eyes owing to an elevated frequency of cell death. We found that if *corp* ^*+*^ was simultaneously overexpressed, the eyes became significantly larger. Furthermore, when we combined the *corp* ^*95B*^ mutant with *GMR>p53* ^*+*^, the eyes were much smaller than produced by *GMR>p53* ^*+*^ alone (Fig 4D). When apoptosis was directly assayed in eye discs of these genotypes, we observed correlated effects, with *corp* ^*+*^ overexpression reducing, and the *corp* mutant increasing cell death (Fig 4Fa-c and 4G). These results may all be accommodated under the hypothesis that Corp antagonizes P53, either by suppressing its apoptotic effects or by negatively regulating P53 itself.

### The level of P53 is inversely correlated with the *corp* ^*+*^ expression level

**Fig 5:**
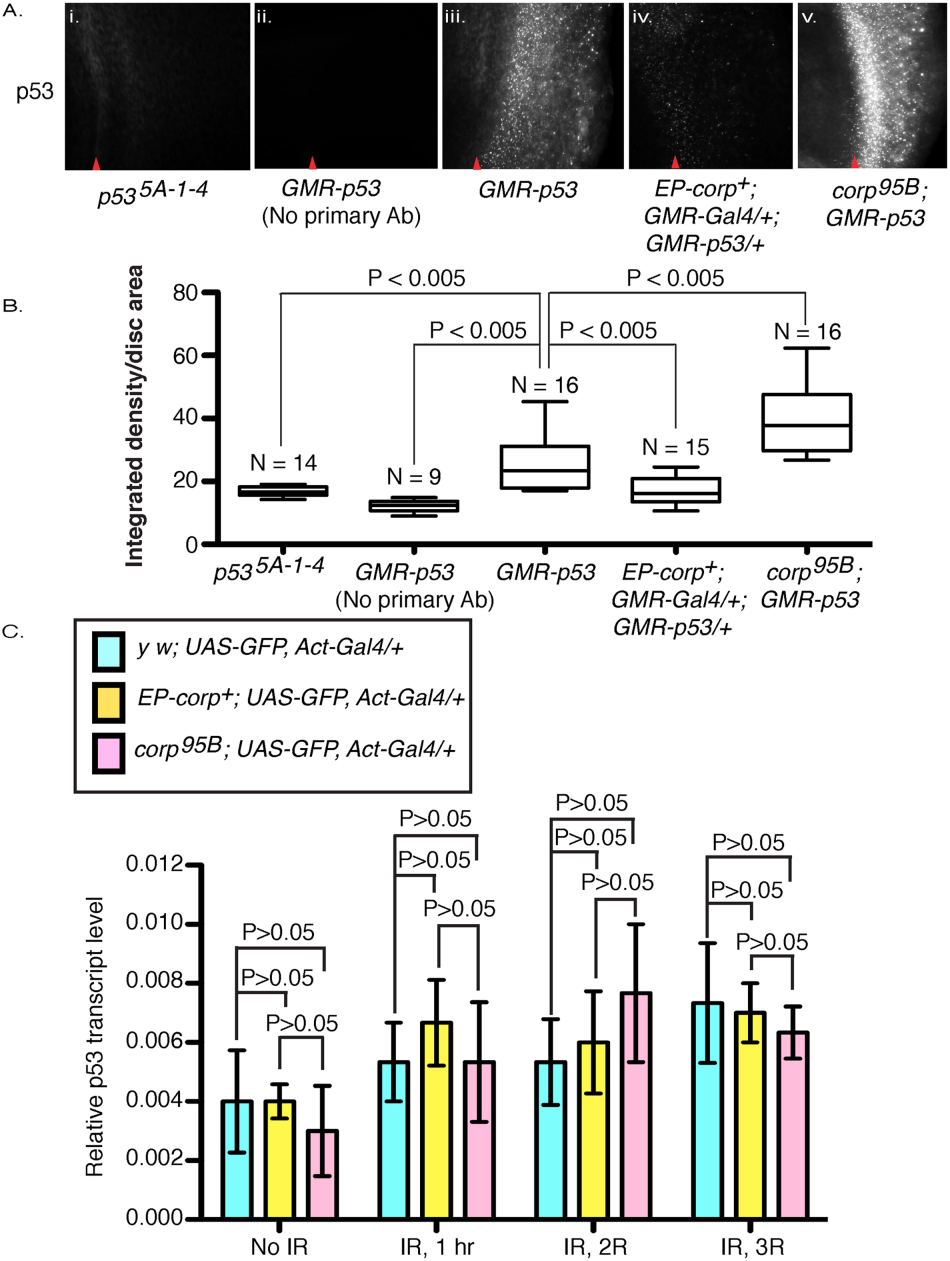
Corp negatively regulates P53 levels and acts similarly to Mdm2. (A) Immunostaining of eye discs by P53 antibody in (i) *p53* ^*5A-1-4*^ mutant larvae and in (ii-v) larvae that overexpress *p53* ^*+*^ under control of the *GMR* promoter, with (ii) no primary antibody staining, (iii) wild-type *corp* ^*+*^ background, (iv) *EP-corp* ^*+*^ overexpression, and (v) *corp* ^*95B*^ mutant. *corp* ^*+*^ overexpression reduces P53 staining, while the *corp* mutant greatly enhances P53 staining. Red arrowheads indicate approximate position of the morphogenetic furrow. Posterior is to the right. Genotypes are as indicated. All images were taken with a 40X objective. (B) Quantitation of P53 immunostaining. P53 staining intensity is measured as integrated density per unit area of eye disc posterior to morphogenetic furrow. N represents the number of eye discs scored. (C) *corp* ^*+*^ does not affect *p53* transcript levels. *p53* mRNA levels were measured by qRTPCR on total cDNA extracts of irradiated and non-irradiated third instar larvae. The graphs represent *p53* mRNA levels with no irradiation and at different time points after irradiation, in control (light blue bars) *corp* ^*+*^ overexpressing (yellow bars) and *corp* ^*95B*^ mutant (pink bars) larvae (as indicated). The larvae were irradiated at 4000 rads and allowed to recover for 1, 2 and 3 hours before cDNA extraction. Y-axis measures copy numbers of *p53* transcript of a particular genotype relative to the copy number of *rpl* transcript of that particular genotype and is denoted as relative *p53* transcript level. Three biological replicates were carried out for each experiment. Data are represented as mean +/- SEM.

To determine how Corp might affect P53 we examined P53 levels in eye discs by immunostaining. The *GMR* promoter was used to overexpress *p53* ^*+*^ behind the morphogenetic furrow, providing an easily detected level of expression, which is absent in a *p53* ^*5A-1-4*^ mutant disc. The *corp* ^*95B*^ mutant eye discs exhibited a significantly higher level of P53 while *EP-corp* ^*+*^ overexpression driven by *GMR-Gal4* reduced the level of P53 (Fig 5A and 5B). Thus, we conclude that Corp is a negative regulator of P53.

To further verify our results, we knocked down *corp* in S2 cells by treating with double-stranded RNA (dsRNA) against *corp* and measured P53 protein levels by Western blot. We found that the quantity of P53 was significantly elevated following *corp* knockdown (S4 Fig), confirming that Corp promotes P53 downregulation.

To determine whether *corp* regulates *p53* at the transcriptional level, we used qRTPCR to measure *p53* mRNA in *corp* mutant or *corp* ^*+*^ overexpressing larvae, with and without irradiation. We found that there are no significant or consistent changes in *p53* mRNA levels between these genotypes (Fig 5C). We conclude that Corp regulation of P53 occurs primarily at the level of translation or protein stability.

### Effect of Corp on expression of *reaper*

The pro-apoptotic *reaper* (*rpr*) gene is a prominent target of P53 following DNA damage [15,21]. If P53 is negatively regulated by *corp* then we expect that *corp* overexpression should also reduce *reaper* induction following IR. To test this, we measured *rpr* mRNA levels by qRTPCR in *corp* ^*+*^-overexpressing and *corp* ^*95B*^ mutant larvae, with and without irradiation. We found that, following IR, *rpr* mRNA levels decrease with *corp* ^*+*^ overexpression and increase in *corp* ^*95B*^ mutants (S5 Fig). Although these results were not significant at the 5% level, they are nonetheless consistent with Corp-mediated downregulation of P53.

### Overexpression of *corp* ^*+*^ suppresses Hid- and Reaper- mediated cell death

Recently, it was shown that the P53 transcriptional targets *hid* and *rpr* act recursively to increase *p53* expression and contribute to the apoptotic program [36]. Given that Corp overexpression results in downregulation of P53, we expect that it should also suppress the apoptotic phenotype caused by *hid* and *reaper* overexpression by attenuating this positive feedback loop. In order to test this prediction, we overexpressed *hid* or *reaper* in the eye under control of the *GMR-Gal4* driver. This produced adults with small eyes owing to cell death in the eye discs. When *corp* ^*+*^ was simultaneously overexpressed the eyes became significantly larger, confirming that Corp interferes with the *hid-* and *reaper*-mediated apoptotic programs (Fig 4E). To confirm that this effect was through inhibition of apoptosis, we directly measured cell death in the eye discs of larvae of the above-mentioned genotypes. We found that rate of apoptosis in *GMR-hid* and *GMR-rpr* eye discs was significantly decreased in a *corp* ^*+*^ overexpressing background (Fig 4Fd-g and 4H).

If *corp* ^*+*^ overexpression rescues the small eye phenotype of *GMR-hid* flies by attenuating the feedback amplification loop through *p53* ^+^, we expect similar rescue from a *p53* mutant. To test this, we measured eye size of *GMR-hid* ^*+*^ *; p53* ^*5A-1-4*^ flies and found that they have significantly larger eyes than *GMR-hid* ^*+*^ control eyes (S6 Fig).

These results are fully consistent with the hypothesis that Corp acts via down-regulation of P53. In this particular case, *corp* ^*+*^-mediated downregulation of P53 blocks the Hid-P53 amplification loop and thereby reduces apoptosis.

### Corp exhibits similarity to the P53-interacting regions of Mdm2

**Fig 6:**
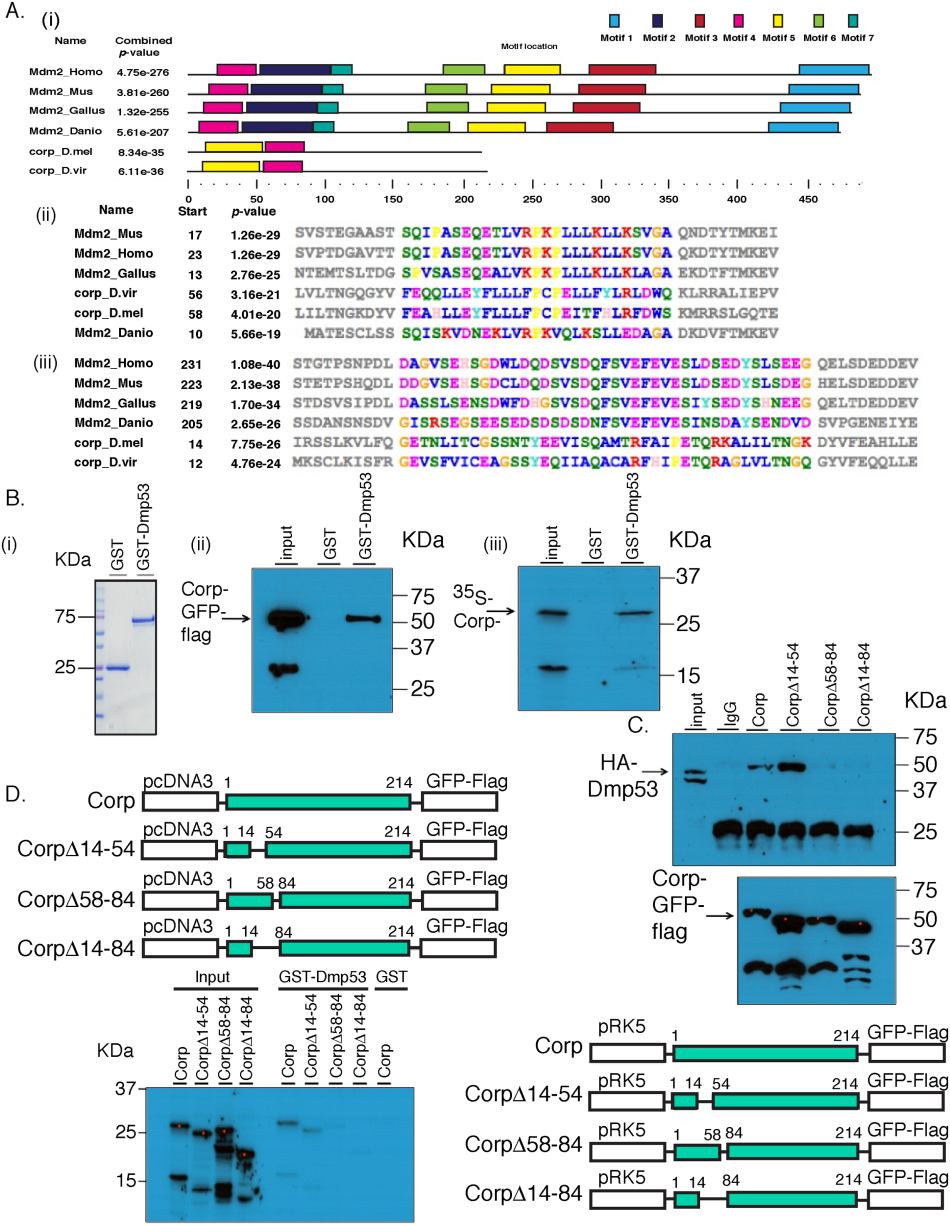
Corp interacts with P53 through a motif shared with Mdm2. (A) Identification of conserved protein motifs between Mdm2 and Corp. MEME was used to identify protein motif similarities between 4 vertebrate Mdm2 orthologs (*H*. *sapiens*, *M*. *musculus*, *D*. *rerio*, *G*. *gallus*) and 2 Corp orthologs from *D*. *melanogaster* and *D*. *virlis*. The software found two motifs conserved between Mdm2 and Corp in these 6 species. (i) A complete map of the 6 proteins indicating the 7 shared motifs, represented by colored rectangles. A scale below indicates the length of the individual proteins in each species and locations of the motifs. Motifs 4 and 5 are found in Corp. (ii) The amino acid sequence of motif 4. The start site indicates the first amino acid residue in that motif. Amino acids in the region of conserved motifs are color-coded [63]. *P-*values that indicate the significance of conservation of each motif were produced by MEME. (iii) The amino acid sequence of the motif 5 region. (B) Corp physically interacts with Drosophila P53 (DmP53). (i) Size distribution of GST and GST-DmP53 recombinant proteins. Coomasie Blue stained gel shows that GST is at 25 KDa and GST-DmP53 is close to 75 KDa. (ii) The results of pull-down from Hela cells expressing Corp-GFP-Flag. Cell lysate was incubated with GST or GST-DmP53 bound to glutathione-agarose beads. Captured proteins were resolved with SDS-PAGE and probed with anti-Flag antibody. The predicted molecular weight of Corp-GFP-Flag is about 55 KDa. The smaller (~33KDa) band on the input lane likely reflects the C-terminal fragment of the fusion protein, which does not interact with GST-DmP53. (iii) *In vitro* synthesized Corp-HA6His (~28KDa) interacts with GST-DmP53. The smaller band likely reflects an N-terminal ^35^S-Methionine containing fragment of Corp, which does interact with DmP53. Input lanes: 0.3% input (ii) and 3% input (iii). (C & D) Corp-Mdm2 Motif 1 (CMM-1, aa 58–84) is required for the interaction between Corp and DmP53. Corp-GFP-FLAG full length and deletion constructs were co-transfected with HA-DmP53 to Hela Cells (C). Following IP with anti-Flag antibody, proteins were resolved on SDS-page and probed with anti-HA (top panel). The amount of Corp was verified with anti-Flag (bottom panel). For each *corp* construct, equal amount of plasmid was used in transfection. The varying abundance of the proteins may result from different transfection efficiency or differential protein stability associated with deletion mutants. Input lane: 0.3% input. (D) *corp* full length and deletion mutants were also cloned into pCDNA3 and used for in vitro transcription and translation in the presence of ^35^S-Methionine. Similar to what we observed via co-IP experiments, deletion mutants without CMM-1 cannot interact with GST-DmP53. Input lane: 5% input.

Mdm2 is the major negative regulator of P53 in vertebrates. However, no homolog of Mdm2 has been found in *Drosophila*. Given that Corp acts in a negative feedback loop on P53, we looked more closely at Corp to see whether any similarities to Mdm2 might be identified. We used the domain analysis tool MEME [37,38] to search for shared protein motifs between four Mdm2 orthologs (*H*. *sapiens*, *M*. *musculus*, *G*. *gallus and D*. *rerio*) and two Corp orthologs from *Drosophila* species (*D*. *melanogaster and D*. *virilis*). It identified seven similar motifs, with motifs 4 and 5 shared by Mdm2 and Corp (Fig 6A). Interestingly, motif 4 appears to correspond to the N-terminal region of Mdm2, the primary P53-interacting domain [39,40]. Motif 5 appears to correspond to an additional P53-binding site on Mdm2 [41]. We named the two motifs Corp and Mdm2 Motif-1 (CMM-1) and CMM-2, respectively.

### Corp physically interacts with P53 through CMM-1

Motivated by the finding of similarities between Corp and Mdm2 in regions of Mdm2 that bind P53, we asked whether *Drosophila* Corp and P53 physically interact. We purified GST-DmP53 using a bacterial expression system. C-terminal-tagged Corp-GFP-Flag was then expressed via transient transfection of HeLa or 293 cells. Cell lysates prepared from these cells were incubated with either GST or GST-DmP53. Corp-GFP-Flag was pulled-down specifically by GST-DmP53 but not by GST (Fig 6Bii), indicating that Corp expressed in mammalian cells interacts with DmP53. This result suggests either that Corp can interact directly with DmP53, or that the complex required for their interaction is conserved in mammalian cells. To further probe this, we tested the interaction between GST-DmP53 and *in vitro* synthesized Corp. We found that GST-DmP53 strongly interacts with *in vitro* synthesized Corp (Fig 6Biii). Together, these results strongly suggest that Corp can interact directly with DmP53.

To test the role of CMM-1 (amino acids 58–84) and CMM-2 (amino acids 14–54) in mediating the interaction between Corp and DmP53, we generated deletion constructs that lack CMM-1 (Δ58–84), CMM-2 (Δ14–54), or both (Δ14–84) (Fig 6C and 6D). The interaction between DmP53 and Corp mutant was assayed via co-immunoprecipitation (Fig 6C) and GST pull-down (Fig 6D). In both systems, proteins that lack CMM-1 have dramatically diminished affinity to DmP53 as compared to full length Corp or Corp(Δ14–54). This indicates that CMM-1, the motif shared with the N-terminal P53-interacting domain of Mdm2, is required for the physical interaction between Corp and Mdm2.

## Discussion

**Fig 7:**
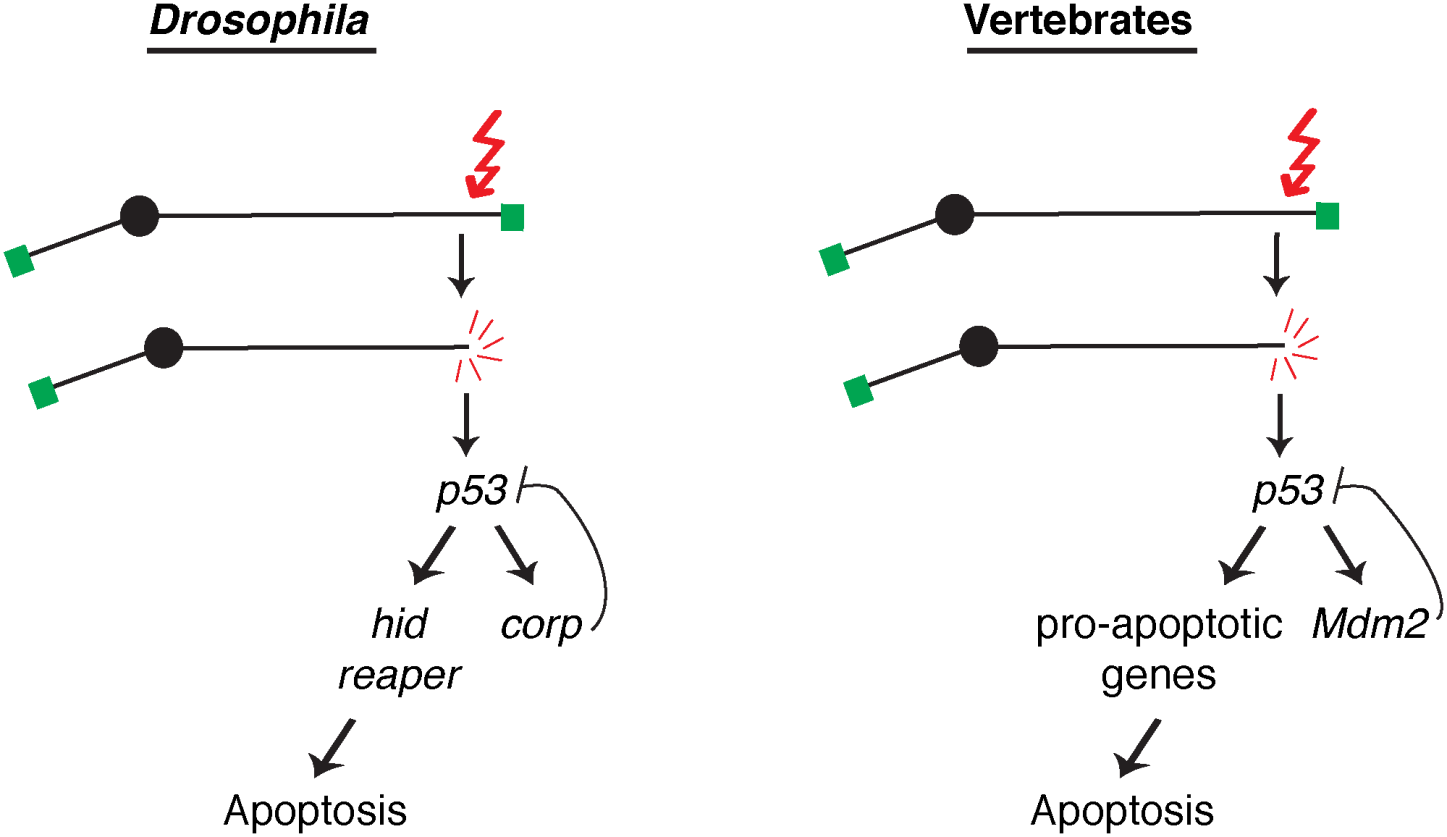
A comparison of competing pathways under P53 control in *Drosophila* and vertebrates. A DNA double-strand break activates the DNA damage response leading to activation of P53. In *Drosophila*, transcription of the well-known pro-apoptotic genes *hid* and *reaper* is activated in one pathway downstream from P53, while transcription of the anti-apoptotic gene *corp* is activated in a second downstream pathway that constitutes a negative feedback loop on P53. These competing pathways are analogous to those found in vertebrates, where P53 promotes apoptosis by inducing a variety of pro-apoptotic genes in one pathway and the P53 negative regulator *Mdm2* in a competing branch. Altering the activity of these competing pathways alters the life or death outcome initiated by the DNA damage signal.

Several studies have identified transcriptional targets of P53 in *Drosophila*. Some of these play important roles in DNA damage repair or in triggering apoptosis [14–17,42,43]. However, the functions of most P53 target genes have yet to be determined. Our discovery that Corp antagonizes apoptosis by negatively regulating P53 is the first demonstration in *Drosophila* that a P53-regulated gene (apart from DNA repair genes) is not solely devoted to apoptosis. Our results show that P53 target genes act in competing pathways, defined by the *hid-* and *reaper-*mediated pro-apoptotic pathway and the *corp-*mediated anti-apoptotic pathway (Fig 7). Increased or decreased expression of *corp* ^*+*^ shifts the balance in favor of survival or death, respectively.

In vertebrates, the major negative regulator of P53 is Mdm2. It binds to P53 and ubiquitinates it, leading to its degradation, and is responsible for restraining P53 activity in unstressed cells. Furthermore, *Mdm2* is also a transcriptional target of P53, and is utilized to turn down the P53 response so that cells that have recovered from the initiating stress, for instance DNA damage, may survive. Though no strict Mdm2 homolog is known in *Drosophila*, our experiments indicate that Corp provides that function. Similar to *Mdm2*, the *corp* gene is a transcriptional target of P53, Corp antagonizes the P53-mediated apoptotic program, P53 levels are inversely correlated with *corp* ^*+*^ expression and Corp physically interacts with P53 through a motif similar to the region of Mdm2 that mediates P53-Mdm2 binding. These similarities strongly support the idea that Corp regulates P53 by a direct physical interaction, thus leading us to propose that Corp is the functional analog of mammalian Mdm2 (Fig 7).

There are significant differences between Corp and Mdm2. Mdm2 is an E3 ubiquitin ligase containing a RING domain [44], but Corp shows no evidence of such a domain. Furthermore, in the mouse *Mdm2* mutations can cause recessive lethality. These mutants may be rescued by the additional mutation of *p53* [45], indicating that lethality results from unrestrained P53 activity. In contrast, the *corp* ^*95B*^ null mutation is not lethal in flies and exhibits no obvious phenotype in the unstressed condition. Corp appears to function only when DNA damage is detected. In *Drosophila* the normal level of P53 expression is insufficient to cause lethality in the absence of Corp unless P53 is activated by upstream kinases.

However, these differences between Corp and Mdm2 may not be as significant as they appear. First, recent findings in mice indicate that the constitutive and induced levels of Mdm2 can be functionally separated. When the P53 Response Element was mutated in the promoter of *Mdm2*, so that Mdm2 was still expressed at a basal level but could no longer be induced to high levels by P53, the resulting mice were viable [46]. Furthermore, when the RING domain of Mdm2 was mutated, so that it no longer functioned as a ubiquitin ligase, but could still interact with its partner Mdmx, the mice were also viable [47]. In both cases the mice were highly sensitive to induced DNA damage, indicating that higher levels of Mdm2 activity are required to recover from DNA damage. Moreover, the latter experiments show that Mdm2 is capable of repressing P53 function without its ubiquitin ligase activity [47]. This may indicate that P53-Mdm2 binding is an ancient mode of regulation, with the ubiquitin ligase activity acquired as a later adaptation. Additionally, recent work has established that Corp physically interacts with the E3 ubiquitin ligase encoded by *hyd* (hyperplastic discs) and with several proteasomal subunits (S7 Fig), suggesting that Corp, like Mdm2, may participate in proteolytic degradation of P53 [48,49].

There is still room for additional explanations for Corp’s phenotypes. If Corp also targeted downstream components of the apoptotic pathway for degradation it might contribute to the phenotypes we observed. Given the existence of a positive feedback loop between downstream pro-apoptotic genes and *p53* [36], Corp might indirectly affect P53 levels by promoting degradation of other components of the apoptotic pathway. However, the physical interaction of Corp and P53 strongly suggests that Corp directly regulates P53, regardless of whether it may also regulate downstream apoptotic components.

Corp is the first reported negative regulator of P53 in *Drosophila* that is also a transcriptional target of P53. Although Bonus and Rad6 have been identified as negative regulators of P53 in *Drosophila* [50,51], neither of them are transcriptional targets of P53, and are thus less similar to Mdm2 than is Corp. Recent experimental findings from others [15,36,52,53], and as reported here, indicate that regulation of P53 is complex, with activation by upstream factors and modulation by positive and negative feedback loops. Further investigation of how these pathways are regulated and how they affect these outcomes should greatly improve our understanding of the many functions of P53.

It remains to be understood what benefit might be provided by Corp. If it is normally desirable to eliminate a cell with unrepaired DNA damage to prevent its proliferation, then what purpose could be served by saving such cells? Unlike mammals, where the function of Mdm2 is needed to restrain P53 in normal cells, Corp appears to function only in cells with damaged genomes. However, previous experiments have shown that in wildtype larvae, many cells with damaged genomes are not eliminated by apoptosis immediately, but rather over a period of a few days [5]. Since *corp* mutants show increased cell death after irradiation, Corp is clearly one factor that restrains the immediate death of cells with damaged genomes. We have often thought it surprising that flies can survive when dicentric chromosomes are formed, and break, in >90% of their cells during development [5,6]. Perhaps if all cells with broken chromosomes immediately succumbed to apoptosis, such flies would not survive. It is easy to imagine that a few remaining survivors, adrift in a sea of dead cells, might not be capable of regenerating a complete imaginal disc. In fact, *corp* mutants survive poorly after widespread induction of *Y* chromosome dicentrics (S1 Table). But, if cells with damaged genomes could be eliminated gradually, it might give the surviving cells a suitable matrix to regenerate a disc. Modulating the rate at which cells are eliminated following lethal damage could be the vital function fulfilled by Corp. [54] recently showed that dying cells signal their neighbors to become resistant to damage-induced death. We would not be surprised to find that this pathway acts through Corp.

## Materials and Methods

### *Drosophila* stocks

All flies were maintained at 25°C on standard cornmeal food. Construction of the *DcY(H1)* and *Dc3(FrTr61A5)1A* chromosomes have been described previously by Kurzhals e*t al*. [20] and *p53* ^*5-A-1-4*^ by Xie *et al*. [55]. We obtained the following stocks from the Bloomington, IN (USA) *Drosophila* stock center: *P{UAS-FLP1*.*D}JD1* (BL 4539), *P{Gal4-ey*.*H}4–8* (BL 5535), *P{EPgy2}CG1632* ^*EY03495*^ (BL 15650), *P{eyFLP*.*N}5* (BL5576), *M{3xP3-RFP*.*attP}ZH-86Fb; M{vas-int*.*B}ZH-102D* (BL 23648), *P{UAS-2xeGFP}AH2* (BL 6874), *nanos-Gal4* [56], *P{GMR-p53*.*Ex}3/TM3*, *Sb*, *Ser* (BL 8417), *GMR-Gal4* [57], *P{GMR-hid}G1/CyO* (BL 5771), *P{GMR-rpr*.*H}S/TM6B*, *Tb* (BL 5773) and *P{Act5C-Gal4}17F01/TM6B*, *Tb* (BL 3954). Two *corp*-RNA*i* stocks: v102751 and v16130 and one *CG1632*-RNA*i* stock: v106107 were obtained from the Vienna Drosophila Resource Center, Vienna, Austria (VDRC).

The following stocks were obtained from Golic lab collections: heat-shock inducible *FLP*, *P{70FLP}10*, *P{UAS-GFP} P{Act-Gal4}/CyO* and *y w; Sp/CyO; nanosGal4 UAS-FLP(95)/TM3*, *Sb*. The *engrailed-Gal4* stock was kindly gifted by Mark Metzstein.

### Plasmids and transgenic constructions

The coding region of *corp* from *Canton* ^*S*^ flies was amplified by PCR with 5’ *NotI* and 3’ *XbaI* overhangs (primers used: Fwd-5’CATATTCGCGGCCGCATGGCCGATATCAGGAGCAG3’ and Rev- 5’CCGCGGGTCTAGACTAGATGCGAATCGAGCGCA3’) and cloned into the pUAST-*w* ^*+*^-attB vector [58]. Vector plasmid was injected in embryos carrying *attP* docking sites on chromosome *3* and *vasa-ΦC31 integrase* on chromosome *4* (BL 23648). *w* ^*+*^ flies were selected for establishing stable transgenic stocks.

The *corp* ^*95B*^ deletion mutation was generated via imprecise excision of the *EY03495 P*-element insertion (Baylor College of Medicine Genome Disruption Project). The DNA break points were identified by PCR amplification (primer sets used: Fwd 1: 5’CCAAGCGAACGCATCGCTG3’, Fwd 2: 5’GAAGAGGTCATCTCCCAAGG3’, Rev1: 5’CTTAGGAACAATGGTTCAACC3’ and Rev2: 5’GCAGCCGAGGTATGGAAATC3’ and sequencing of genomic DNA obtained from the homozygous mutant.

### DNA sequencing

Sequencing of *corp* ^+^ from the genomic region in five different genotypes, *y w*, *w* ^*1118*^, *EP-corp* ^*+*^, *Canton* ^*S*^ and *v; Sco/Cy; ry*, was carried out by the Core Facilities, University of Utah.

### Eye photographs

Eye photographs were taken using a Nikon D200 digital camera and processed in Adobe Photoshop.

### Quantitative reverse transcriptase PCR

Total RNA was extracted from 12–15 adults or third instar larvae using Trizol Reagent (Sigma Aldrich, MO), treated with DNaseI (Fermentas, PA) and cDNA was synthesized using RevertAid First Strand cDNA synthesis kit (Thermo Scientific, PA) according to manufacturer’s protocol. 1μl of cDNA was used per reaction in triplicates for performing qRT-PCR experiment using Maxima SYBR green/Fluorescein qPCR Master Mix (Fermentas, PA) or PerfeCTa SYBR Green FastMix (Quanta Biosciences, MD) in an iQ-PCR machine (Bio-Rad, CA). Relative quantification of mRNA levels was calculated using the standard curve method. Relative copy numbers of each gene of interest (X) was calculated by normalizing cDNA levels of X over cDNA levels of Ribosomal Protein L32. Primers that were used are: Fwd-corp: 5’ GCAGCCGAGGTATGGAAATC 3’; Rev-corp: 5’ AAGCCGAGGGTCAGAAGG 3’; Fwd-p53: 5’ GCCGCCTCCTTAATCATGCC 3’; Rev-p53: 5’ GCCGAGACTGCGACGACTC 3’; Fwd-rpr: 5’ CCAGTTGTGTAATTCCGAACGA 3’;[59] Rev-rpr: 5’ GGATCTGCTGCTCCTTCTGC 3’;[59] Fwd-rpl: 5’ CCGCTTCAAGGGACAGTATC3’; Rev-rpl: 5’ ATCTCGCCGCAGTAAACG 3’.

### Irradiation

15–18 wandering third instar larvae were collected in clean 10 mm petri plates and irradiated at 4000 rads using a TORREX120D X-ray generator (Astrophysics Research Corp, CA) or a Mark I irradiator (J. L. Shepherd & Associates, CA). These larvae were returned to fresh food and incubated at 25°C until further experimental treatments.

### Eye size measurement

For determining eye sizes, the left eye of each fly was measured along the anterio-posterior axis (A) and the dorso-ventral axis (B), using a digital filar micrometer (Lasico, CA). These two measurements were used to calculate the area of an ellipse (i.e., Π x A/2 x B/2), as the area of the eye, which was then normalized to the mean area of wildtype (*w* ^*1118*^ or *y w*) eyes, and was represented as a fraction of wildtype eye size.

### Germline fragment chromosome transmission assay

Flies were allowed to lay eggs and transferred to fresh vials every day. Embryos were collected for 24 hrs, heat-shocked at 38°C for one hour in a circulating water bath and then immediately returned to 25°C. After eclosion, the males were collected and singly mated to 2–3 *y w* females and their progeny were scored. Alternatively, *nosGal4* was used to drive *UAS-FLP* in the male germline.

### Imaginal disc staining and fluorescence microscopy

Wing and eye imaginal discs were dissected from third instar larvae and stained with TUNEL or P53 antibody.

#### TUNEL staining

TUNEL staining was performed using Apoptag Red *In Situ* Apoptosis Detection Kit (#S7165, Chemicon International). Briefly, dissected imaginal discs were fixed in 4% paraformaldehyde, rinsed twice in PBT_W_ (0.3% Tween-20 in 1X PBS) for 5 minutes/rinse, post-fixed in 2:1 EtOH/1X PBS, rinsed again as before and then incubated with equilibration buffer, TdT enzyme and anti-digoxigenin Rhodamine congujate antibody in subsequent steps, according to manufacturer’s protocol. Finally, the discs were mounted in Vectashield (Vector Laboratories Inc., CA) and photographed in Z projection. The images were exported as 8 bit TIFF files and analyzed in Adobe Photoshop. Fluorescence intensity of TUNEL staining was measured, using ImageJ software, as the total pixel intensity (referred to as Integrated density or InD) within each disc divided by the area of that disc. For wing discs the area of the entire wing disc was considered, or divided into anterior and posterior compartments, as appropriate. For eye discs, where gene expression was driven with *GMR*, only the region behind the morphogenetic furrow was considered for measuring InD.

#### P53 immunostaining

Third instar larvae were dissected in 1X PBS and fixed in 4% paraformaldehyde for 15 minutes at room temperature. They were washed in PBT_X_ (0.3% Triton-X in 1X PBS) twice for 30 minutes each, followed by 1 hour blocking in 5% BSA in PBT_X_. Next, they were incubated overnight at 4°C in primary antibody, p53-7A4 (DSHB, University of Iowa, IA) at 1:10 concentration in 5% BSA. The discs were then rinsed twice with PBT_X_, 30 minutes each and once in blocking buffer for 1 hour and finally incubated with Alexa Fluor 568 goat anti-mouse secondary antibody (Invitrogen, OR) at 1: 1000 concentration in 5% BSA for two hours. Finally, they were washed twice with PBT_X_ as before, mounted in Vectashield (Vector Laboratories Inc., CA) and photographed in Z projection. All images were taken at 100 ms shutter speed and at neutral density 6. Minimum and maximum intensity range was set to 200–550 for all discs, and images were exported as 8-bit TIFF files. Fluorescence was analyzed in Adobe Photoshop. P53 staining intensity was measured, using ImageJ software, as InD divided by the eye disc area, with only the region behind the morphogenetic furrow considered (Fig 5B). For visual representation (Fig 5A), gamma was set to 0.65 and contrast was increased to 100 to reduce background staining and improve visualization of differences between genotypes.

### dsRNA synthesis and purification

To synthesize double stranded RNA for RNA interference experiments with cultured cells, PCR products not more than 700 base pairs, were made of the cDNA of interest flanked by T7 RNA polymerase sites at both ends. After gel purification of the PCR product, it was used as template for *in vitro* transcription for 6 hours at 37°C in a circulating water bath in 5–6 replicates of 20 μl reaction each for better yield using Ambion Megascript T7 Transcription Kit (Life Technologies), according to manufacturer’s protocol. Then, the reactions were pooled together in a microcentrifuge tube and extracted with phenol-chloroform and chloroform. Finally, dsRNA was precipitated with ispropanol, dissolved in DEPC-treated H_2_O and quantified in a Nanodrop 1000 spectrophotometer (Thermo Scientific).

Primers used for obtaining PCR products were: Fwd_T7corp: 5’TTAATACGACTCACTATAGGGAGAATGGCCGATATCAGGAGCAG3’; Rev_T7corp: 5’TTAATACGACTCACTATAGGGAGACTAGATGCGAATCGAGCGCA3’; Fwd_T7p53: 5’TTAATACGACTCACTATAGGGAGAAGATCCAGGCGAACACGCTG3’; Rev_T7p53: 5’TTAATACGACTCACTATAGGGAGAGGCTTCCGGCACGGACTTG3’; Fwd_T7Pav: 5’TTAATACGACTCACTATAGGGAGAACAACTGCTCTTGGCAGATACC3’; Rev_T7Pav: 5’TTAATACGACTCACTATAGGGAGAAAATCCGTAACGAAACTAACCG3’.

### S2 cell culture

S2 cells were cultured at 25°C in Schneider’s *Drosophila* Medium (Invitrogen) with 10% heat inactivated fetal bovine serum (HyClone) and 1X Antibiotic-Antimycotic (Invitrogen). Cells were passaged into fresh medium every 3–4 days and were discarded after passage 25 (P25).

### dsRNA treatment of S2 cells and western blot

The dsRNA treatment protocol was performed as described [60]. Cells were passaged on day 0 at the rate of 2 x 10^6^ cells/ml. On day 1 they were washed and seeded in 24-well plates at 800 μl/well. 15 μg of dsRNA added to each well. The plates were then returned to the 25°C incubator. On day 4–5, cells were re-seeded in 6 well plates at 2 ml/well and retreated with 30 μg of dsRNA. As a control of dsRNA uptake rate, cells were treated with Pavarotti dsRNA, which makes them large and multinucleate[61,62]. On day 6–7, cells were collected, lysed and processed for Western blot.

S2 cells, with or without dsRNA treatment, were irradiated at 4000 rads to observe any elevation/change in P53 levels from unirradiated cells, with or without dsRNA treatment. No significant changes in P53 levels were observed following irradiation, so treated cells were grouped and categorized as (I) no dsRNA control group and (II) + dsRNA experimental group for quantification.

The Western results were pulled from 3 independent experimental sets in the *p53*-dsRNA treatment experiment and 5 independent experimental sets in the cor*p*-dsRNA treatment experiment. Out of the 3 *p53*-dsRNA experimental sets, 1 was following irradiation at 4000 rads and allowing 4 hours recovery before cell lysis. Likewise, out of the 5 *corp*-dsRNA experimental sets, 2 were following irradiation. Relative P53 levels in each experiment were calculated by normalizing total P53 protein level to total β–tubulin level.

#### Western blotting

S2 cells were collected and lysed in RIPA buffer containing protease inhibitor (Thermo Scientific, IL) to a final concentration of 1X. Protein concentration was measured by BCA assay (Thermo Scientific, IL) and cell lysates were mixed with sample buffer and β–mercaptoethanol to a final concentration of 1X before loading onto a 10% SDS gel at equal concentrations. Western blotting was carried out following standard procedure. Antibodies used: mouse monoclonal anti-*Drosophila* P53 (# sc-74573, Santa Cruz Biotechnology; as used by Chen *et al*.[51]) at 1:1000 concentration and mouse monoclonal anti-*Drosophila* β–tubulin (E7, Developmental Studies Hybridoma Bank, University of Iowa, IA) at 1:10,000 concentration. After incubation with fluorescent goat anti-mouse secondary antibody (# 926–68020, Li-COR Biosciences) at 1:10,000, the membranes were scanned on an infrared Odyssey scanner (LI-COR Biosciences).

### GST and GST-DmP53 purification

The open reading frame of DmP53 was cloned into pGEX and transformed to BL21 DE3 cells. The expression was induced by addition of IPTG to a final concentration of 0.1mM in LB/Ampicillin media. Bacterial cells were harvested 4 hours following the induction and resuspended in ice-cold STE buffer (10mM Tris-HCl pH8.0, 150mM NaCl, 1mM EDTA and protease inhibitors) with 1.5% Sarcosyl. Cells were then lysed with sonication and subsequently incubated with STE containing 1% Triton X-100 for 30 minutes. Insoluble proteins were removed by centrifugation at 16,000g for 5 minutes. Supernatant was then incubated overnight with 50% slurry of glutathione-agarose beads at 4°C. The beads were pelleted by centrifuge at 100g and washed 4 more times with 10 ml of ice-cold PBTP (PBS with 0.1% Triton X-100 and protease inhibitors). Washed beads were then resuspended in PBTP with 0.01% sodium azide.

### Expression of Corp-GFPFlag in HeLa cells

15ug of pRK5-*corp-gfp-flag* plasmid DNA was transfected to 2 million HeLa cells with calcium phosphate. Cells were lysed on plate with 1ml RIPA buffer at 48 hours following transfection.

### In vitro synthesis of Corp-HA6His

Corp-HA6His was cloned into pCDNA3. In vitro synthesis were carried out using the TnT Coupled Reticulocyte Lysate Systems (Promega, Catalog number L4611) following manufacturer’s instructions.

### GST pull-down assays

For *in vitro* synthesized protein, 1 ug GST or GST-DmP53 bound to Glutathione-agarose beads was incubated with 5 ul of synthesized protein in 500ul Binding buffer (50mM Tris-HCl, pH 8.0; 2mM EDTA; 150mM NaCl; 0.1% NP40; 20uM ZnCl_2_; 10mM MgCl_2_; protease inhibitors) containing BSA (0.2ug/ul). Following 1 hour incubation at RT and 1 hour incubation at 4°C, the beads were washed 4 times with Binding buffer. Beads were then pelleted at 100g, re-suspended and boiled in 30 ul sampling buffer, and resolved on SDS-PAGE gel. Following electrophoresis, the gel was fixed in (Isopropenol:dH_2_O:Acitic acid = 25:65:10) for 30 minutes and incubated in the Amplify Fluorographic Reagent (GE Healthcare, NAMP100) for 1 hour. The gel was then vacuum dried and processed for autoradiography with an intensify screen at -80°C.

For cellular extract, 1 ug GST or GST-DmP53 bound to Glutathione-agarose beads was first incubated for 30 minutes in 500ul binding buffer with 0.2ug/ul BSA. 500 ul cell lysate was then added and incubated at 4°C for overnight. Following incubation the beads were washed 4 times with 1 ml of RIPA buffer and pelleted by centrifugation at 100g for 5 minutes. Beads were then resuspended in 30ul sampling buffer and resolved on SDS-PAGE gel. Western analysis was performed with anti-Flag M2 antibody (Sigma, F1804).

### Graphical methods and statistical analyses

Construction of graphs and calculations of statistical significance were performed using Prism 5.0 (Graphpad). In box-and-whisker plots the ends of whiskers represent 5^th^ and 95^th^ percentiles, top and bottom of the boxes represent 25^th^ and 75^th^ percentile and the horizontal line in the box represents the median, i.e., 50^th^ percentile. The Mann-Whitney test was used in Figs 2, 3, 4, 5 and S1, S3, S6 Figs; Unpaired t-test in S4C and S5 Figs; and, Paired t-test in S4D Fig.

### Software

The MEME tool used for searching motif similarity is publicly available software (http://meme.nbcr.net/meme/). Images were quantitatively analyzed using IMAGE J software from National Institutes of Health (http://imagej.nih.gov/ij/index.html) and images were processed using Adobe Photoshop. All line diagrams were composed using Adobe Illustrator.

## Supporting Information

S1 Fig *EP-corp* ^*+*^ induces *corp* ^*+*^ overexpression. *corp* mRNA levels were measured by qRTPCR on total cDNA extracts from *Actin-Gal4 EP-corp* ^*+*^ or *y w* control adults. The Y-axis indicates *corp* transcript levels normalized to the *Rpl32* transcript. Data is represented as mean +SEM. N represents the number of biological replicates of each experiment. Statistical significance was calculated by the Mann-Whitney test. Although *corp* expression was ~15X higher when the *EP-corp* ^*+*^ was driven, the difference was not significant at the 5% level.
(TIF)

S2 Fig Deletion mapping in the *corp* ^*95B*^ mutant. (A) The *corp* genomic region on the *X* chromosome and *corp* transcripts (RA and RB; adapted from FlyBase: http://flybase.org/reports/FBgn0030028.html). Orange shading denotes the protein coding regions. The blue arrowhead indicates the site of the *EY03495 EPgy2*. Imprecise excision of the *EY03495* element produced the *corp* ^*95B*^ allele. The extent of the *corp* ^*95B*^ deletion was determined by genomic sequencing. The nucleotide coordinates of the deleted region are X: 8,269,857–8,271,938 (Genome Release 6). Two sets of primers, Fwd1, Rev1 and Fwd2, Rev2 were used for PCR amplification of *corp* genomic region. (B,C) Visualization of PCR results. The *corp* genomic region was amplified either by Fwd1, Rev1 or by Fwd 2, Rev2 primer pairs in four different genotypes, run in the four lanes on the gel, marked 1 through 4: (1) *y w*; (2) *corp* ^1^ (not used in this work); (3) *corp* ^*95B*^; and (4) *corp* ^2^ (not used in this work). M1 is a 1 kb ladder and M2 is the 100 bp ladder. The vertical red arrow indicates the lane 3, which lacks any PCR product and corresponds to the *corp* ^*95B*^ template. The horizontal red arrowhead points to the size expected in the *y w* control.
(TIF)

S3 Fig Altered *corp* function only affects eyes after dicentric chromosome induction. (A) The range of eye phenotypes observed in the BARTL assay, reproduced from Fig 2A. The distribution of eye sizes when (B) *H1* dicentrics are produced in the presence of uninduced *EP-corp* ^*+*^; (C) *EP-corp* ^*+*^ is induced in *B* ^*+*^ flies without dicentric induction; (D, E) *EP-corp* ^*+*^ is present, either uninduced (D) or induced (E) in *B* ^*S*^ flies without dicentric induction; (F) *corp* ^*95B*^ is introduced into *B* ^*S*^ background without dicentric induction; (G) *corp* ^*95B*^ is introduced into *B* ^*+*^ flies without dicentric induction; (H) *H1* dicentrics are produced in *XXY(H1)* females; (I) *EP-corp* ^*+*^ is overexpressed and dicentric chromosomes are induced in *XXY(H1)* females; (J) dicentric chromosome formation is induced on chromosome 3 (*Dc3*); and (K) *EP-corp* ^*+*^ is overexpressed and chromosome *3* dicentrics are induced. *N* represents the number of fly eyes scored for each genotype. The *P* value in S3-B represents comparison with the wildtype control, Fig 2B. The other two *P* values in S3-I and S3-K represent comparison with the graphs immediately above them, i.e., S3-H and S3-J respectively.
(TIF)

S4 Fig P53 protein level increases in *corp* knockdown cells. (A, B) Western Blots of protein extracts from S2 cells. The left lanes represent untreated cells (wildtype) while the right lanes represent cells treated with (A) *p53*-dsRNA or (B) *corp*-dsRNA. β–tubulin is the loading control. The efficiency of dsRNA uptake by cells was approximately 50%. (C, D) Quantitation of P53 protein levels. Protein level is quantified by measuring the integrated density of the area of the desired band of the protein (P53 and β–tubulin) on the blot divided by the band area (InD/ band area). P53 level is represented in Y axis as the ratio of InD/ band area of P53 normalized to that of β–tubulin. *N* represents the number of experimental sets. Data are represented as mean +SEM.
(TIF)

S5 Fig Corp affects transcriptional activation of *rpr* following irradiation. *rpr* mRNA levels were measured by qRTPCR on total cDNA extracts of irradiated and non-irradiated third instar larvae. The graph represents *rpr* mRNA levels with no irradiation and at different time points after irradiation, in control (blue bars) *corp* ^*+*^ overexpressing (cyan bars) and *corp* ^*95B*^ mutant (magenta bars) larvae (as indicated). Full genotypes are indicated in the inset. The larvae were irradiated at 4000 rads and allowed to recover for 2 or 3 hours before cDNA extraction. The Y-axis indicates *rpr* transcript quantitation relative to the *rpl* transcript internal control. *rpr* level alterations in corp overexpressing and corp mutant larvae, compared to controls, are not statistically significant. Three biological replicates were carried out for each experiment. Data are represented as mean +SEM.
(TIF)

S6 Fig The *p53* mutant suppresses the cell-death phenotype caused by *hid* ^*+*^ overexpression. *GMR* drives overexpression of *p53* ^*+*^ in the eye to produce very small eyes that are alleviated by *p53* ^*5A-1-4*^ mutants. Y-axis represents the fraction of a wildtype eye. N, the number of eyes measured for quantification.
(TIF)

S7 Fig Protein interactions of Corp in *Drosophila melanogaster*. This figure, taken from FlyBase (http://flybase.org/cgi-bin/get_interactions.html?items=FBgn0030028&mode=ppi), shows proteins that have been identified as physically interacting with Corp. Nine of these 19 interactors are proteasome subunits, and one is an E3 ubiquitin ligase (hyd).
(TIF)

S1 Table Viability of *corp* mutants after induction of *Y* chromosome dicentrics. ^*a*^ *y w/DcY*, *H1; 70FLP10/SM1*, *Cy* males X *y w* females ^*b*^ *y w/DcY*, *H1; 70FLP10/SM1*, *Cy* males X *y w corp* ^*95B*^ females *P* values calculated with contingency test.
(DOCX)
