# Supplementary figures and images for: Corp Regulates P53 in Drosophila melanogaster via a Negative Feedback Loop
Source: PLoS Genet. 2015 Jul 31;11(7):e1005400. doi: 10.1371/journal.pgen.1005400 (PMC4521751; doi:10.1371/journal.pgen.1005400)

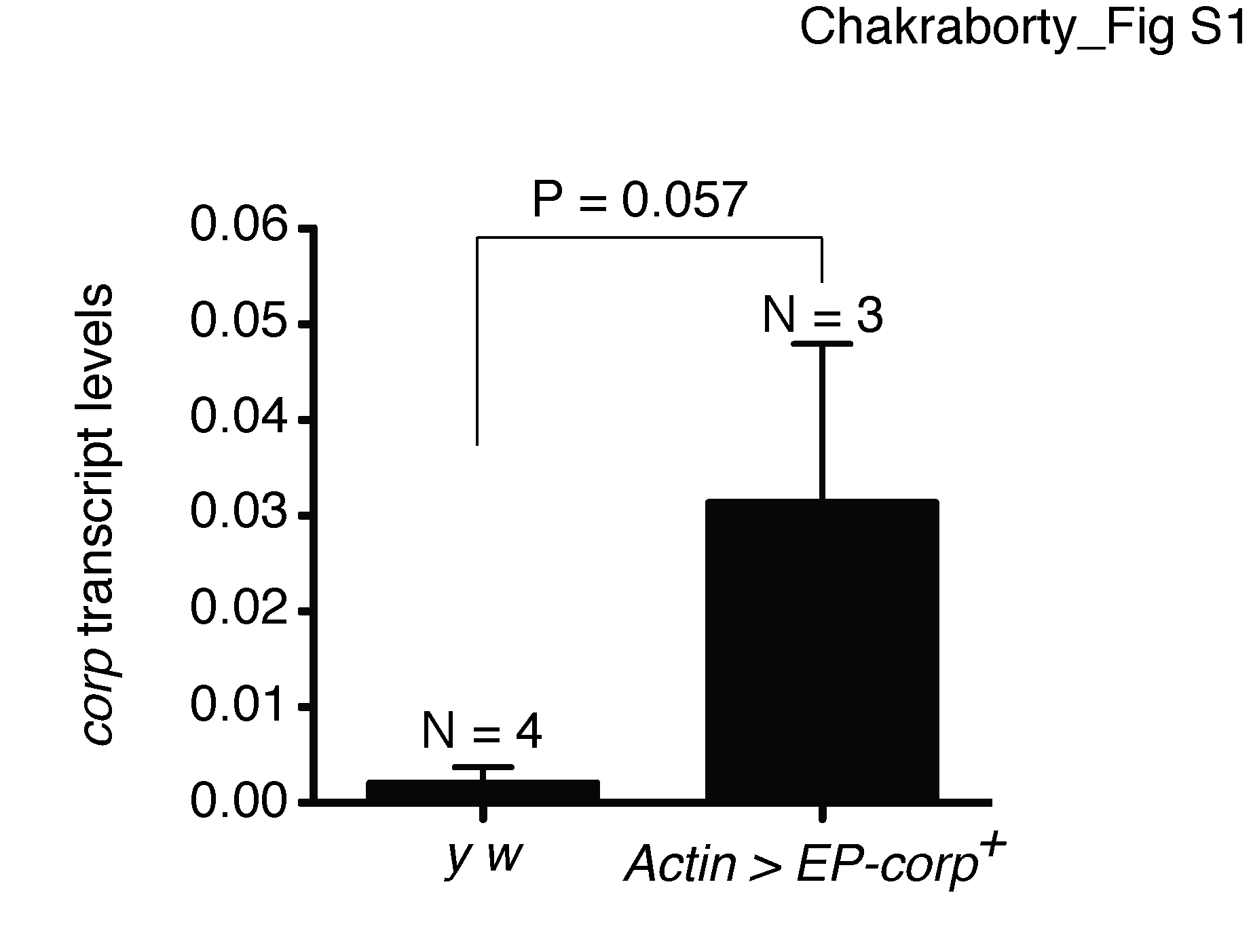

Supplement: S1 Fig — corp mRNA levels were measured by qRTPCR on total cDNA extracts from Actin-Gal4 EP-corp + or y w control adults. The Y-axis indicates corp transcript levels normalized to the Rpl32 transcript. Data is represented as mean +SEM. N represents the number of biological replicates of each experiment. Statistical significance was calculated by the Mann-Whitney test. Although corp expression was ~15X higher when the EP-corp + was driven, the difference was not significant at the 5% level. (TIF) [file pgen.1005400.s001.tif]

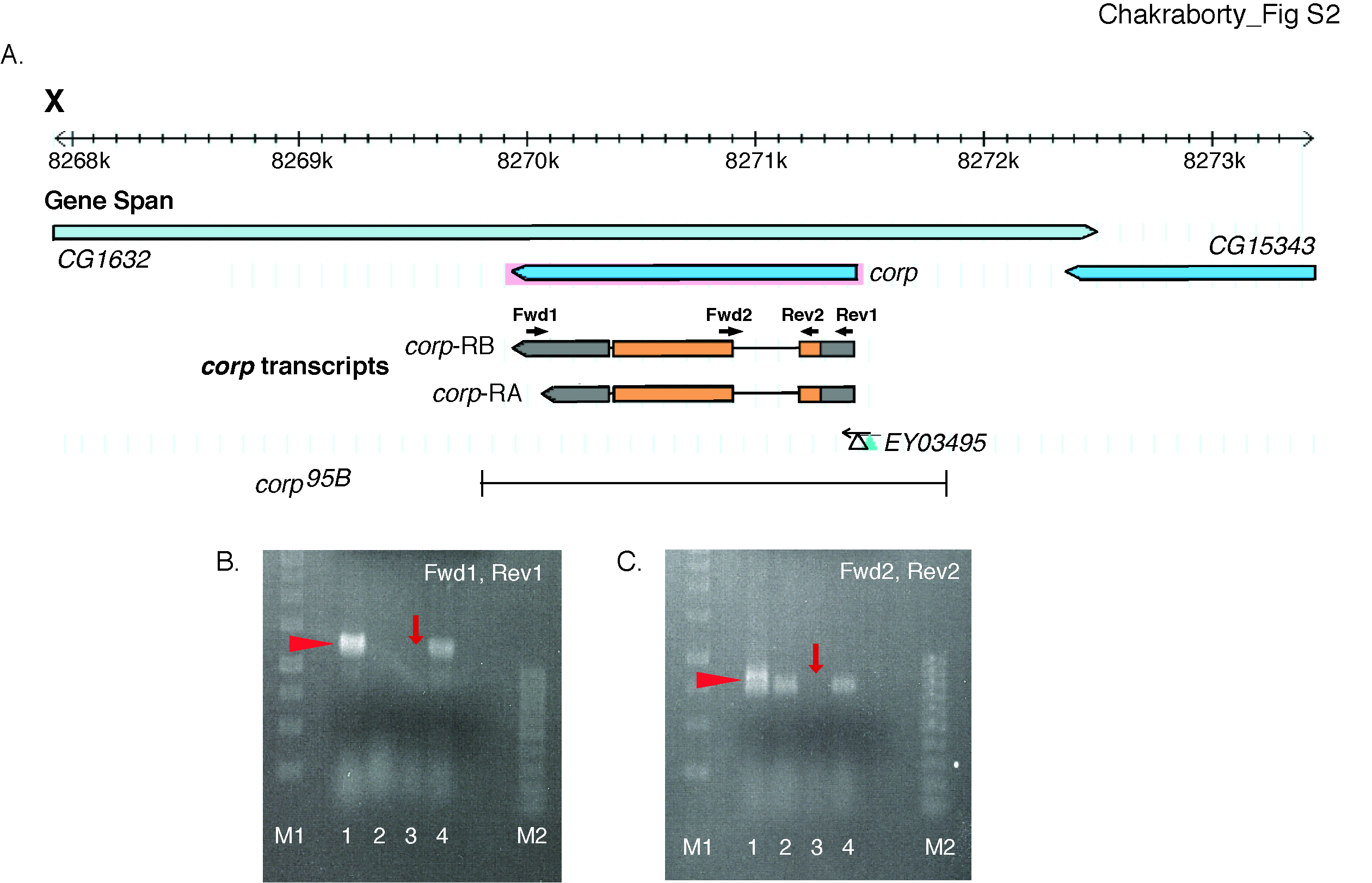

Supplement: S2 Fig — (A) The corp genomic region on the X chromosome and corp transcripts (RA and RB; adapted from FlyBase: http://flybase.org/reports/FBgn0030028.html). Orange shading denotes the protein coding regions. The blue arrowhead indicates the site of the EY03495 EPgy2. Imprecise excision of the EY03495 element produced the corp 95B allele. The extent of the corp 95B deletion was determined by genomic sequencing. The nucleotide coordinates of the deleted region are X: 8,269,857–8,271,938 (Genome Release 6). Two sets of primers, Fwd1, Rev1 and Fwd2, Rev2 were used for PCR amplification of corp genomic region. (B,C) Visualization of PCR results. The corp genomic region was amplified either by Fwd1, Rev1 or by Fwd 2, Rev2 primer pairs in four different genotypes, run in the four lanes on the gel, marked 1 through 4: (1) y w; (2) corp 1 (not used in this work); (3) corp 95B; and (4) corp 2 (not used in this work). M1 is a 1 kb ladder and M2 is the 100 bp ladder. The vertical red arrow indicates the lane 3, which lacks any PCR product and corresponds to the corp 95B template. The horizontal red arrowhead points to the size expected in the y w control. (TIF) [file pgen.1005400.s002.tif]

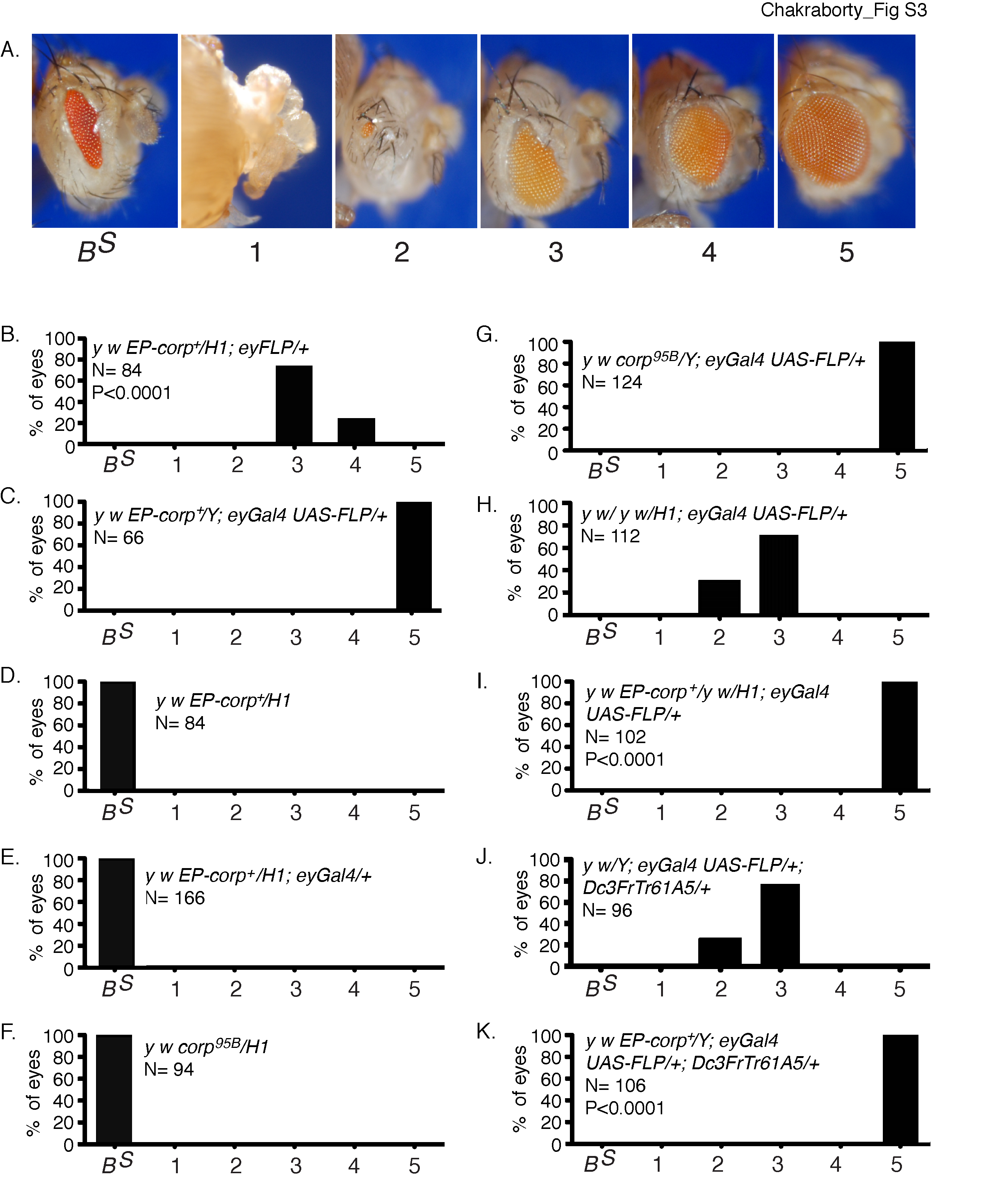

Supplement: S3 Fig — (A) The range of eye phenotypes observed in the BARTL assay, reproduced from Fig 2A. The distribution of eye sizes when (B) H1 dicentrics are produced in the presence of uninduced EP-corp +; (C) EP-corp + is induced in B + flies without dicentric induction; (D, E) EP-corp + is present, either uninduced (D) or induced (E) in B S flies without dicentric induction; (F) corp 95B is introduced into B S background without dicentric induction; (G) corp 95B is introduced into B + flies without dicentric induction; (H) H1 dicentrics are produced in XXY(H1) females; (I) EP-corp + is overexpressed and dicentric chromosomes are induced in XXY(H1) females; (J) dicentric chromosome formation is induced on chromosome 3 (Dc3); and (K) EP-corp + is overexpressed and chromosome 3 dicentrics are induced. N represents the number of fly eyes scored for each genotype. The P value in S3-B represents comparison with the wildtype control, Fig 2B. The other two P values in S3-I and S3-K represent comparison with the graphs immediately above them, i.e., S3-H and S3-J respectively. (TIF) [file pgen.1005400.s003.tif]

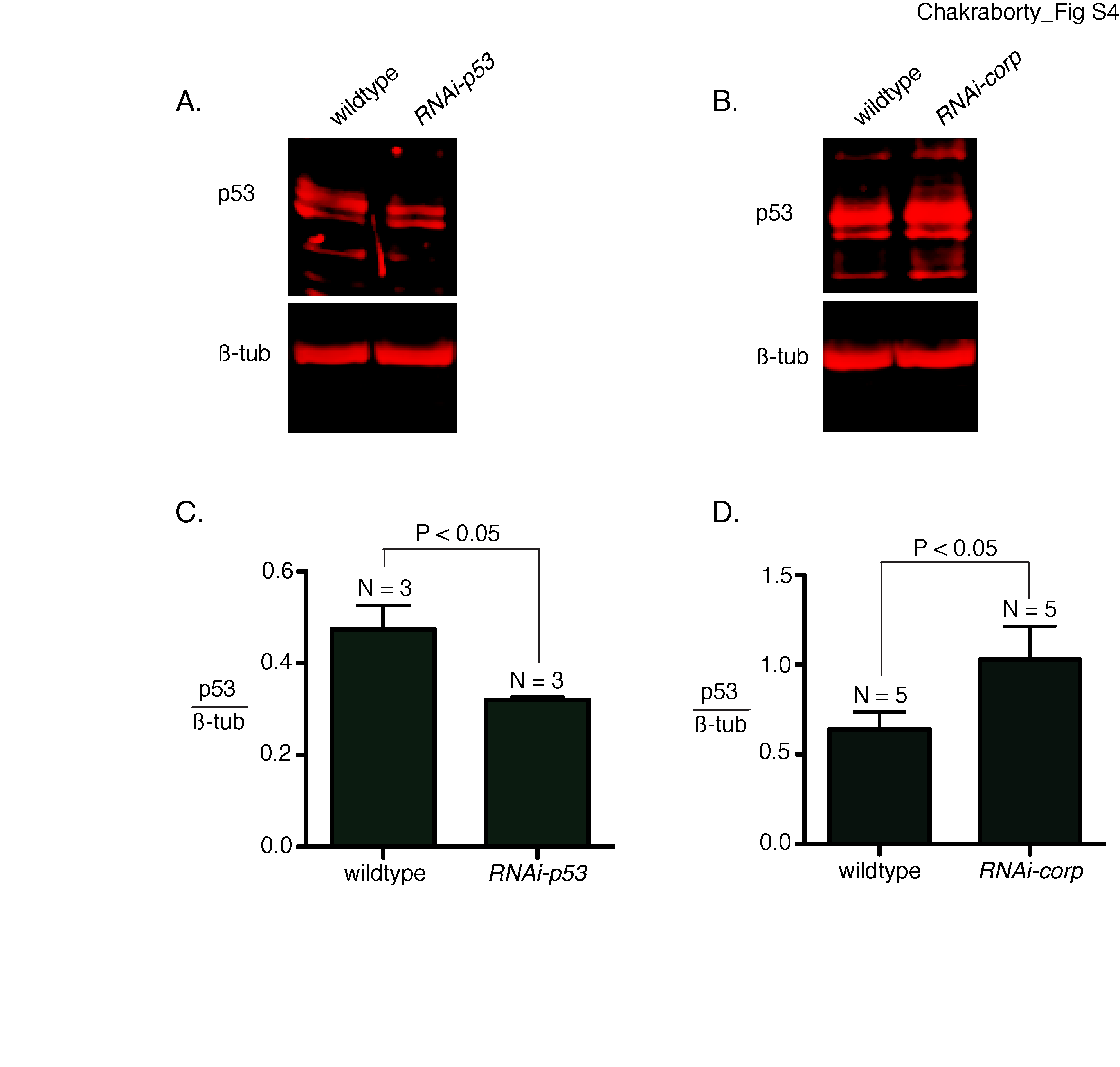

Supplement: S4 Fig — (A, B) Western Blots of protein extracts from S2 cells. The left lanes represent untreated cells (wildtype) while the right lanes represent cells treated with (A) p53-dsRNA or (B) corp-dsRNA. β–tubulin is the loading control. The efficiency of dsRNA uptake by cells was approximately 50%. (C, D) Quantitation of P53 protein levels. Protein level is quantified by measuring the integrated density of the area of the desired band of the protein (P53 and β–tubulin) on the blot divided by the band area (InD/ band area). P53 level is represented in Y axis as the ratio of InD/ band area of P53 normalized to that of β–tubulin. N represents the number of experimental sets. Data are represented as mean +SEM. (TIF) [file pgen.1005400.s004.tif]

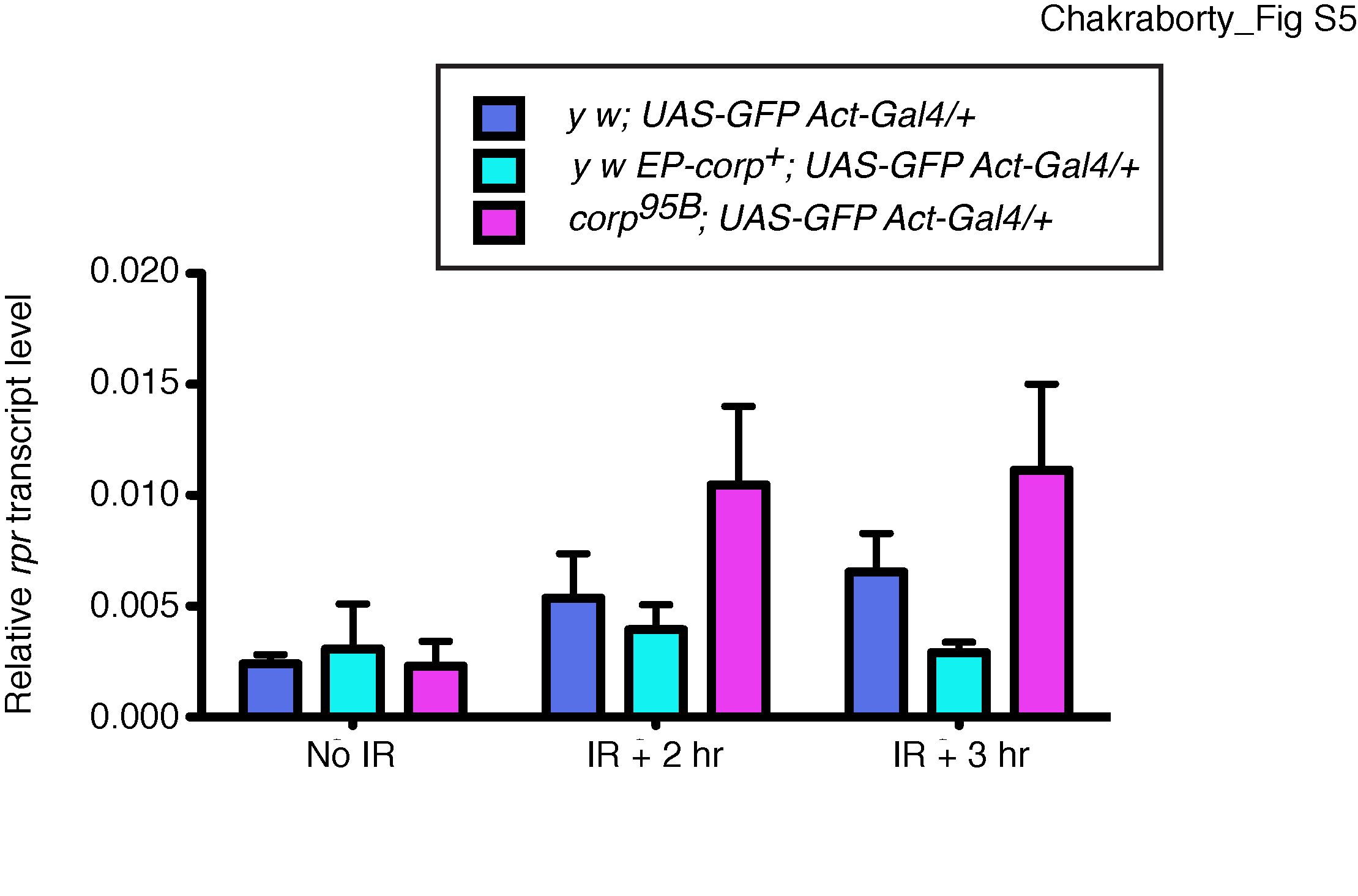

Supplement: S5 Fig — rpr mRNA levels were measured by qRTPCR on total cDNA extracts of irradiated and non-irradiated third instar larvae. The graph represents rpr mRNA levels with no irradiation and at different time points after irradiation, in control (blue bars) corp + overexpressing (cyan bars) and corp 95B mutant (magenta bars) larvae (as indicated). Full genotypes are indicated in the inset. The larvae were irradiated at 4000 rads and allowed to recover for 2 or 3 hours before cDNA extraction. The Y-axis indicates rpr transcript quantitation relative to the rpl transcript internal control. rpr level alterations in corp overexpressing and corp mutant larvae, compared to controls, are not statistically significant. Three biological replicates were carried out for each experiment. Data are represented as mean +SEM. (TIF) [file pgen.1005400.s005.tif]

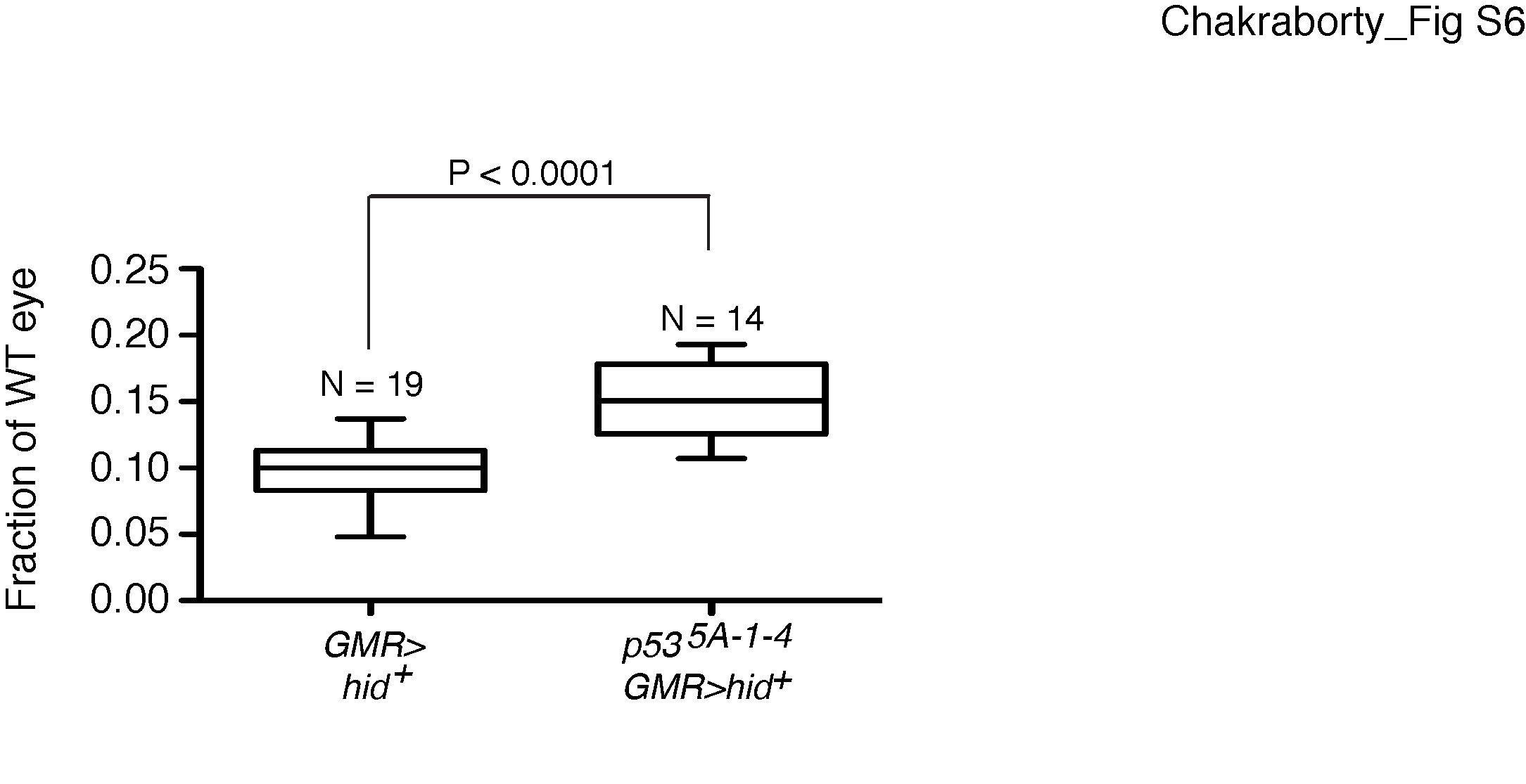

Supplement: S6 Fig — GMR drives overexpression of p53 + in the eye to produce very small eyes that are alleviated by p53 5A-1-4 mutants. Y-axis represents the fraction of a wildtype eye. N, the number of eyes measured for quantification. (TIF) [file pgen.1005400.s006.tif]

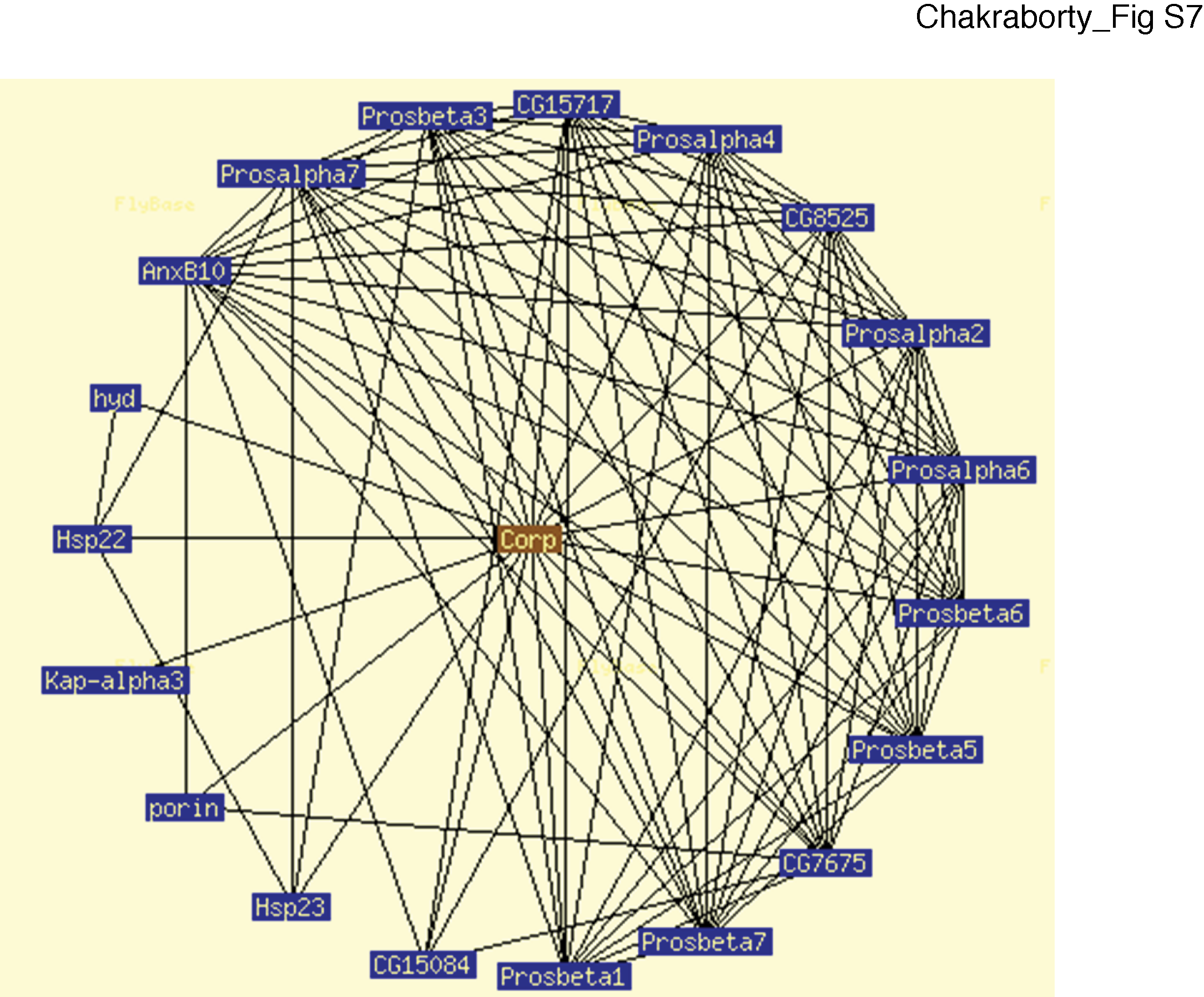

Supplement: S7 Fig — This figure, taken from FlyBase (http://flybase.org/cgi-bin/get_interactions.html?items=FBgn0030028&mode=ppi), shows proteins that have been identified as physically interacting with Corp. Nine of these 19 interactors are proteasome subunits, and one is an E3 ubiquitin ligase (hyd). (TIF) [file pgen.1005400.s007.tif]
