# Supplementary material for: Corp Regulates P53 in Drosophila melanogaster via a Negative Feedback Loop
Source: PLoS Genet. 2015 Jul 31;11(7):e1005400. doi: 10.1371/journal.pgen.1005400 (PMC4521751; doi:10.1371/journal.pgen.1005400)
Supplement: S1 Table — a y w/DcY, H1; 70FLP10/SM1, Cy males X y w females b y w/DcY, H1; 70FLP10/SM1, Cy males X y w corp 95B females P values calculated with contingency test. (DOCX) [file pgen.1005400.s008.docx]

**S1 Table.**

| **Treatment**  **condition** | **Genotypes** | **sons** | | **daughters** | |
| --- | --- | --- | --- | --- | --- |
|  |  | + FLP | -FLP | + FLP | -FLP |
| -HS  (*P =* 0.12) | *^a^ corp^+^* | 142 | 135 | 164 | 168 |
|  | *^b^ corp^95B^* | 88 | 88 | 146 | 110 |
| +HS  (*P* < 0.0001) | *^a^ corp^+^* | 133 | 179 | 163 | 168 |
|  | *^b^ corp^95B^* | 30 | 138 | 124 | 120 |
